# Transcriptional responses of human intestinal epithelial HT-29 cells to spore-displayed p40 derived from *Lacticaseibacillus rhamnosus* GG

**DOI:** 10.1186/s12866-022-02735-3

**Published:** 2022-12-22

**Authors:** Soo Ji Kang, Jeong A Moon, Do Yeong Son, Kwang Won Hong

**Affiliations:** Department of Food Science and Biotechnology, College of Life Science and Biotechnology, Dongguk University, Goyang-si, 10326 Republic of Korea

**Keywords:** Spore surface display, p40 protein, HT-29 cells, RNA-sequencing, Differentially expressed genes

## Abstract

**Backgrounds:**

The aims of this study were to construct spore-displayed p40, a *Lacticaseibacillus rhamnosus* GG-derived soluble protein, using spore surface display technology and to evaluate transcriptional responses in human intestinal epithelial cells.

**Results:**

p40 was displayed on the surface of *Bacillus subtilis* spores using spore coat protein CotG as an anchor protein. Effects of spore-displayed p40 (CotG-p40) on gene expression of intestinal epithelial cell line HT-29 were evaluated by transcriptome analysis using RNA-sequencing. As a result of differentially expressed gene (DEG) analysis, 81 genes were up-regulated and 82 genes were down-regulated in CotG-p40 stimulated cells than in unstimulated cells. Gene ontology enrichment analysis showed that CotG-p40 affected biological processes such as developmental process, metabolic process, cell surface receptor linked signaling pathway, and retinoic acid metabolic process. Gene-gene network analysis suggested that 10 DEGs (*EREG, FOXF1, GLI2, PTGS2, SPP1, MMP19, TNFRSF1B, PTGER4, CLDN18,* and *ALDH1A3*) activated by CotG-p40 were associated with probiotic action.

**Conclusions:**

This study demonstrates the regulatory effects of CotG-p40 on proliferation and homeostasis of HT-29 cells. This study provided comprehensive insights into the transcriptional response of human intestinal epithelial cells stimulated by CotG-p40.

**Supplementary Information:**

The online version contains supplementary material available at 10.1186/s12866-022-02735-3.

## Background

Probiotics are live microorganisms that can confer a health benefit on the host when administered in appropriate amounts [1]. Despite many health benefits, probiotics have some limitations because they are live microorganisms. The effects of probiotics vary depending on the gut microbiome compositions of the host and on strains or doses of probiotic bacteria ingested [2]. Because probiotic bacteria are greatly affected by factors such as pH, temperature, moisture, and air, there are concerns about their viability and stability during storage or processing as well as in the human gastrointestinal (GI) tract [3]. To overcome these limitations, the research on probiotics are shifting focus to proteins derived from probiotic bacteria [4]. They not only mimic the probiotic activity of probiotic bacteria, but also have several advantages such as a specific mechanism of action and ease of storage and production [5, 6]. Thus, they are considered as a safe alternative to compensate limitations associated with live probiotics. Moreover, many researchers have been attempting to develop novel probiotics with desirable effects using probiotics engineering technology that comprehends metabolic engineering and synthetic biology [2, 7, 8].

*Lacticaseibacillus rhamnosus* GG (LGG) is one of the most commonly used Gram-positive probiotics strains isolated from healthy human intestines [9]. LGG can help treat diseases such as diarrhea, GI disorders, and atopic dermatitis [10–12]. As one of secreted proteins from LGG, p40 plays a role in the probiotic functionality of LGG [13]. p40 protein also is known to carry cysteine, histidine-dependent aminohydrolase/peptidase (CHAP) domain that exhibits peptidoglycan hydrolase (PGH) activity [14]. p40 protein suppresses disruption of barrier function and cytokine-induced apoptosis of intestinal epithelial cells by activating epidermal growth factor (EGF) receptor [15]. The activated EGF receptor also enhances proliferation, migration, and survival of intestinal epithelial cells [16]. Another study has revealed that p40 protects intestinal epithelial cells against injuries and maintain their homeostasis [17]. Although roles of p40 in intestinal epithelial cells are well understood at the protein level, its roles at the gene expression level are unclear [16–18].

*Bacillus subtilis* is a spore-forming Gram-positive bacterium [19]. It also has good safety records in that it has been used as an additive in human and animal preparations as probiotics [20]. Spore surface display technology offers many functional and economic advantages. *B. subtilis* spores are comprised of core, cortex, and more than 70 coat proteins including inner coat and outer coat proteins [21]. Since spores have a rigid structure, they can withstand heat, radiation, and chemicals in harsh environments such as industrial process or GI tract [22]. This unique structure of spore provides the enhanced stability to the protein displayed on the spore surface [21, 22]. These properties are extremely useful for oral delivery. In addition, only cultivation and centrifugation were required to prepare the spore displayed proteins. Spores can be obtained at a high rate (up to 10^10^ spores/mL) through flask culture and can be stored for a long period of time after purification [23]. Due to these advantages, spore surface display has been applied in several fields, such as whole-cell biocatalyst and vaccine development [24–27]. In our previous study, another LGG-derived p75 protein was displayed on the spore surface and its effects on transcriptional response of human intestinal epithelial cells was evaluated [28, 29].

In this study, we aimed to construct spore-displayed p40 (CotG-p40) by displaying LGG-derived p40 protein on the surface of *B. subtilis* spore using CotG as an anchor protein. The effect of CotG-p40 on transcriptional response of HT-29 cells is evaluated at the gene expression level by RNA-sequencing (RNA-seq). Analysis methods such as differentially expressed genes (DEGs), gene ontology (GO) enrichment, and gene-gene interaction network analysis were used to comprehend effects of CotG-p40 on gene expression of HT-29 cells.

## Results

### Construction of spore-displayed p40

**Fig. 1:**
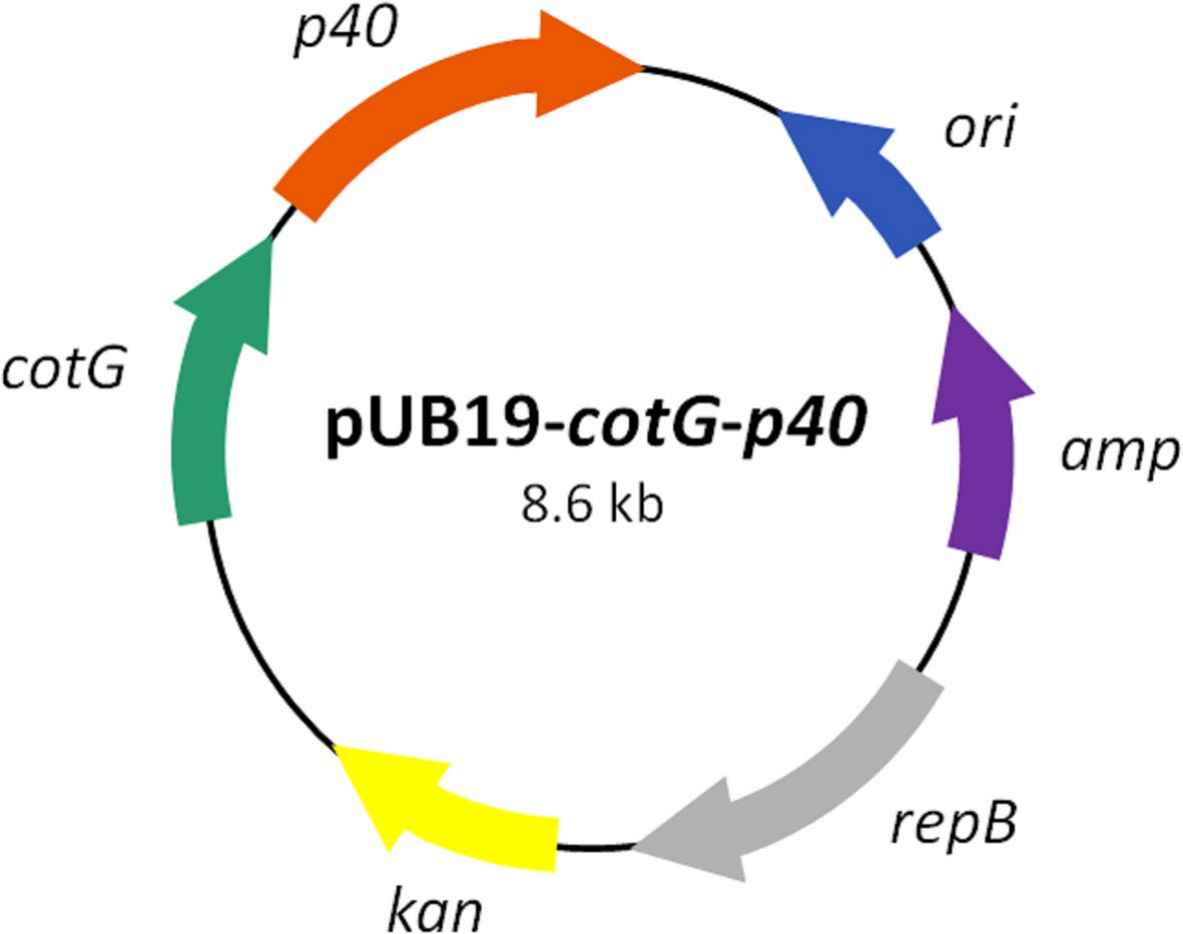
Plasmid diagram of recombinant plasmid. pUB19-*cotG-p40* includes antibiotic markers (*kan*, kanamycin; *amp*, ampicillin), replication origin (*ori*), and replication protein B (*repB*). *cotG* represents the spore coat protein CotG encoding gene of *Bacillus subtilis*, and p40 represents the p40 protein encoding gene of *Lacticaseibacillus rhamnosus* GG

To construct the recombinant plasmid pUB19-*cotG*- *p40*, the *cotG* gene from the outer coat protein CotG of *B. subtilis* 168 and the *p40* gene from *L. rhamnosus* GG were amplified and overlapped. The overlapped *cotG*-*p40* fragment was inserted into pUB19 vector. The pUB19 plasmid was digested with *Not*I and *Mlu*I and ligated. A flexible linker (Gly-Gly-Gly-Gly-Ser) was inserted between the C-terminus of CotG and the N-terminus of p40 to provide flexibility of structural domain movements. The construction of plasmid was verified by restriction enzymes digestion and polymerase chain reaction (PCR) methods. The recombinant plasmid was named pUB19-*cotG*-*p40* and a diagram of its structure is presented in Fig. 1.

### Expression of spore-displayed p40

**Fig. 2:**
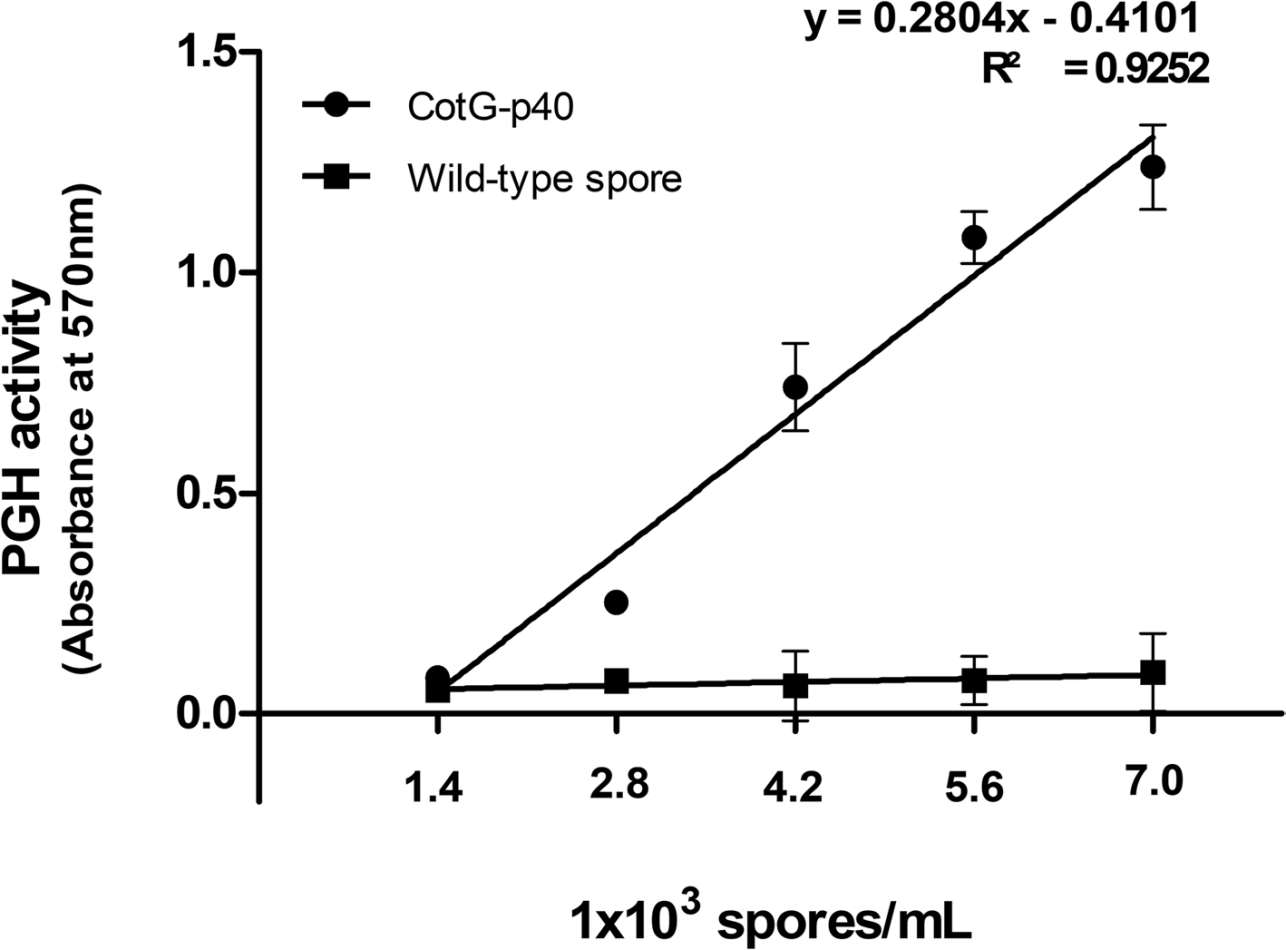
Determination of the peptidoglycan hydrolase activity of CotG-p40. After treatment of peptidoglycan with different concentrations of CotG-p40 (●) and wild-type spore (■) at 37 °C for 15 min, the absorbance of each sample was measured at 570 nm. All tests were performed in triplicate, and the data are presented as mean ± standard deviation

The expression of p40 on the spore surface was verified by a ninhydrin test in that p40 exhibits PGH activity. Peptidoglycan (PG) was exposed to various concentrations of CotG-p40 (1.4, 2.8, 4.2, 5.6, and 7.0 × 10^3^ spores/mL). The PG degradation levels were determined by the ninhydrin test. As presented in Fig. 2, the absorbance increased linearly as CotG-p40 concentration increased (*R*^2^ = 0.9252). This result showed that the PGH activity of p40 protein was well maintained even when displayed on the spore surface. Therefore, it is considered that the fusion of p40 and CotG protein does not affect the biological activity of p40 protein. On the other hand, the PGH activity of wild-type spores was not observed (Fig. S1).

### Thermal and pH stability of spore-displayed p40

**Fig. 3:**
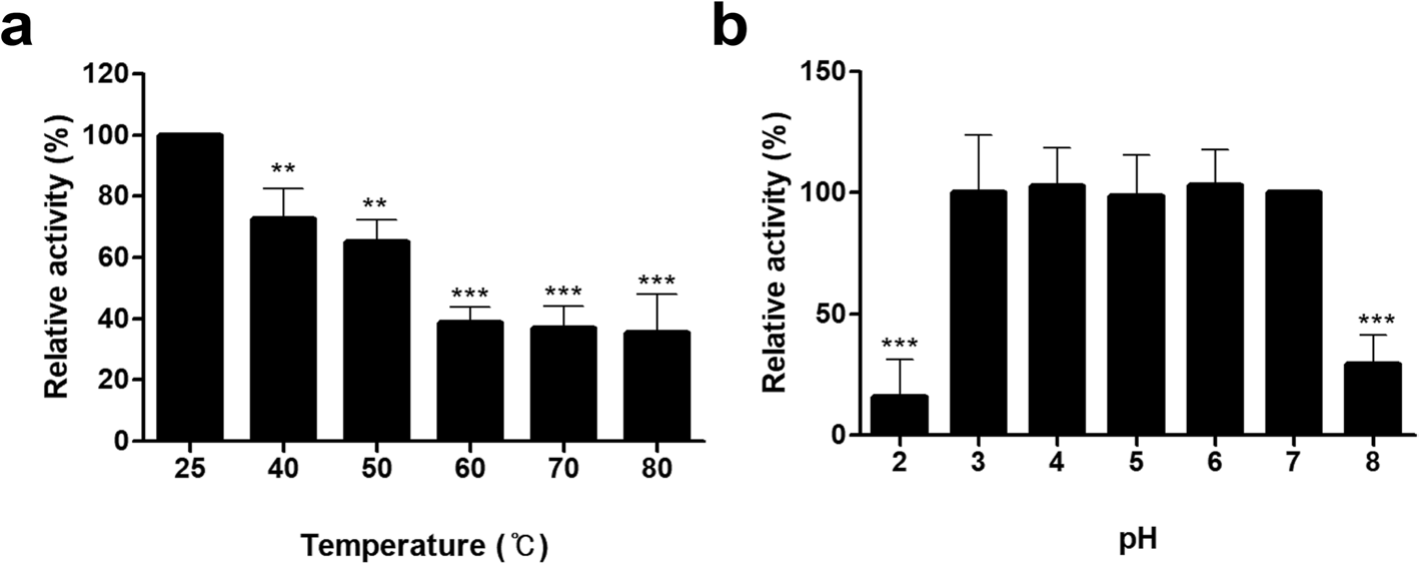
Relative peptidoglycan hydrolase activity of CotG-p40 after heat (**a**) and pH (**b**) treatments. Relative activity was calculated by defining its activity at 25 °C and pH 7 as 100%. All tests were performed in triplicate, and the data are presented as mean ± standard deviation. Statistical analysis was performed by an unpaired two-tailed *t*-test. Asterisks (*) indicate a significance difference from the control (* *p* < 0.05, ** *p* < 0.01, *** *p* < 0.001)

The stable expression of PGH activity of CotG-p40 was determined under various temperature and pH conditions. The PGH activity of CotG-p40 measured at room temperature (25 °C) and pH 7 was used as a control. As shown in Fig. 3a, when the temperature increased from 40 °C to 80 °C, the PGH activity of CotG-p40 decreased gradually. However, CotG-p40 still retained its initial activity more than 70 and 65% at 40 and 50 °C. CotG-p40 exhibits high thermostability retaining more than 35% of its activity even at a high temperature of 80 °C. The pH stability is also tested under various pH conditions. As presented in Fig. 3b, CotG-p40 maintained its original PGH activity at a wide range of pH 3–7 (*p* > 0.05). However, the PGH activity of CotG-p40 was greatly reduced to 15% at pH 2 strongly acidic and 30% at pH 8 a relatively weak alkaline.

### RNA-sequencing and data analysis

To investigate transcriptional responses, RNA-seq was performed for CotG-p40 treated- and wild-type spore treated- human intestinal epithelial HT-29 cells. All samples were treated for 3 h. Cells treated with PBS instead of spores were used as a control. Before functional analyses were conducted, quality control analysis was carried out for raw data obtained from RNA-seq. Clean reads were obtained after removing low-quality data and adapter sequences from raw data. As a result, 62,200,250, 80,150,244, and 77,090,466 clean reads were obtained from cells treated with CotG-p40, wild-type spores, and control, respectively, with clean ratios more than 98%. The average Q30 quality score of these three samples was above 95% and the percentage for mapped reads was over 97% (Table S1).

### Differentially expressed genes in HT-29 cells stimulated with CotG-p40

**Fig. 4:**
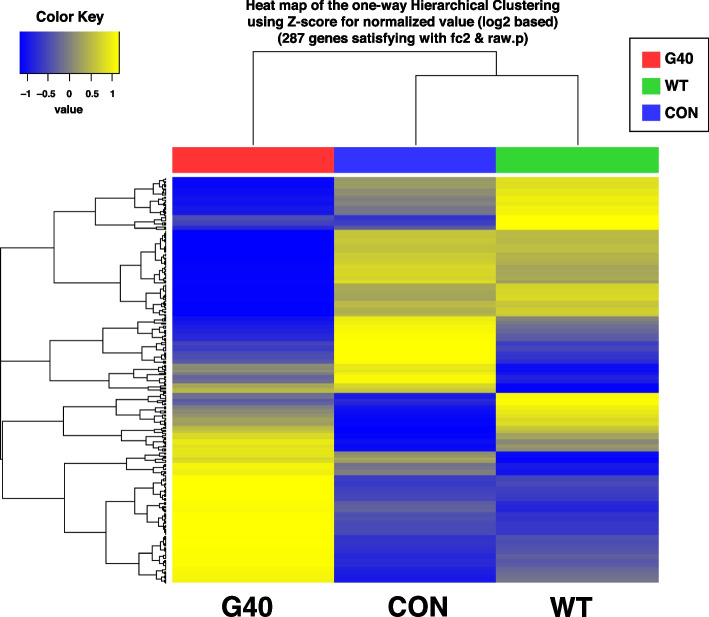
Analysis of hierarchical clustering. Heat map shows expression levels of DEGs in three samples. Each column represents three different samples of CotG-p40 treated cells (G40), control cells (CON), and wild-type spore treated cells (WT). Each row represents DEGs. High expression level is indicated by “yellow” and lower expression level is indicated by “blue”

**Fig. 5:**
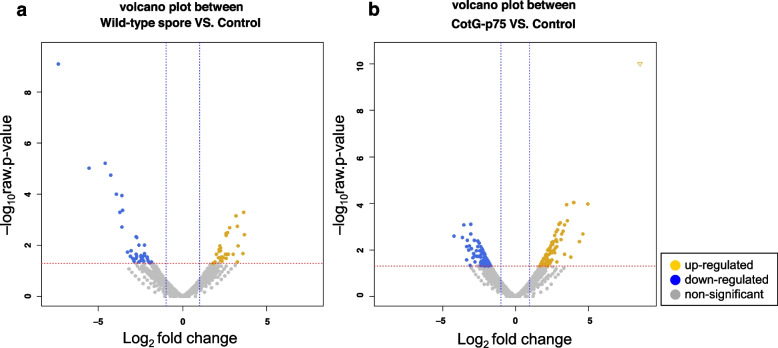
Volcano plot of all differentially expressed genes between two samples. Fold change values are plotted on the x-axis and negative log10 *p*-values are plotted on the y-axis. Up-regulated genes are indicated by “yellow”. Down-regulated genes are indicated by “blue”. Genes showing no significant difference are indicated by “gray”. a, Volcano plot between wild-type spore-treated cells and control; b, Volcano plot between CotG-p40 treated cells and control

DEG analysis was performed to determine transcriptome changes in HT-29 cells treated with CotG-p40. Genes with |fold change| ≥ 2 and raw *p*-value < 0.05 were considered as DEGs. A total of 163 DEGs were detected between the CotG-p40 treatment group and the control group, including 81 up-regulated and 82 down-regulated genes. A total of 75 DEGs were detected between the wild-type spore treatment group and the control group, showing 36 up-regulated and 39 down-regulated genes (Table S2). To confirm the similarity of expression, DEGs between samples were represented by heat maps as shown in Fig. 4. Results showed that DEGs in control and wild-type spore-stimulated cells were similar in their expression, but different from those in CotG-p40-stimulated cells. As shown in the volcano plot of Fig. 5, there were more DEGs between the control and CotG-p40 treated group than between the control and wild-type spore treated group. These results confirmed that transcriptome changes in HT-29 cells were mediated by p40 displayed on the spore surface.

Among genes activated by CotG-p40, DEG analysis results of genes associated with the probiotic function of LGG are shown in Table 1. fold change values of *ZO-1*, *CLDN*, *OCLN*, *PTGS2*, *MMP19*, *ADAM17*, *HB-EGF*, *MUC2*, and *APRIL* genes are shown. Expression levels of *PTGS2* and *MMP19* were increased by 3.2 and 4.87 times, respectively, confirming that gene expression patterns of CotG-p40 and p40 were partially consistent. The results of DEG analysis for all comparison groups, CotG-p40 stimulated cells vs. control, CotG-p40 stimulated cells vs. wild-type spore stimulated cells, and wild-type spore stimulated cells vs. control, are provided in Table S2 − S4 in supplementary file, respectively.

**Table 1:** Fold change values of genes related to intestinal function improvement of *Lacticaseibacillus rhamnosus* GG in CotG-p40 stimulated HT-29 cells

Gene symbol	Description	Fold change
*ZO-1 (TJP1)*	Tight junction protein 1	− 1.37
*CLDN1*	Claudin 1	−1.11
*OCLN*	Occludin	1.02
*PTGS2*	Prostaglandin-endoperoxide synthase 2	3.20
*MMP19*	Matrix metallopeptidase 19	4.87
*ADMA17*	ADAM metallopeptidase domain 17	1.24
*HB-EGF*	Heparin-binding EGF-like growth factor	2.44
*MUC2*	Mucin 2, oligomeric mucus/gel-forming	1.24
*APRIL*	A proliferation-inducing ligand	−1.00

### Gene ontology enrichment analysis and gene-gene interaction network analysis

**Fig. 6:**
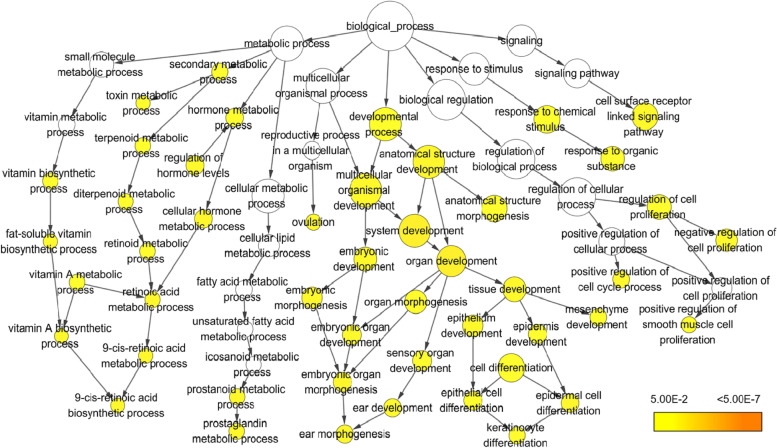
Gene ontology (GO) analysis for DEGs between CotG-p40 treated cells and control. The color of each node represents the enrichment level of each GO term. The size of each node represents the number of genes that map to the category represented by each node. The color saturation of the node represents the significance (*p*-value) of the category represented by each node

GO enrichment analysis was performed to analyze biological functions associated with DEGs. Gene ontology is largely divided into three categories: biological process, cellular component, and molecular function. According to the analysis, there were 46 GO terms with corrected *p*-value < 0.05, all of which belonged to biological process (Table S3). Among these 46 GO terms, the top 20 GO terms based on the corrected *p*-value are presented in Table 2. The interaction between all GO terms is presented as a network as shown in Fig. 6 using BiNGO. As a result, most genes activated by CotG-p40 were annotated in organ development (GO:0048513), system development (GO:0048731), anatomical structure development (GO:0048856), tissue development (GO:0009888), epithelial development (GO:0060429), and multicellular organismal development (GO:0007275). These are subordinate to the developmental process (GO: 0032502). Results also confirmed that many DEGs were involved in GO terms such as cell surface receptor linked signaling pathway (GO:0007166) under signaling (GO:0023052) and retinoic acid metabolic process (GO: 0042573) associated with the synthesis of vitamin A and retinoic acid under metabolic process (GO: 0008152). The results of GO enrichment analysis for all comparison groups, CotG-p40 stimulated cells vs. control, CotG-p40 stimulated cells vs. wild-type spore stimulated cells, and wild-type spore stimulated cells vs. control, are provided in Table S5, Table S6 (Fig. S2), and S7 (Fig. S3) of the supplementary file, respectively.

**Table 2:** Top 20 gene ontology (GO) terms of differentially expressed genes between CotG-p40 treated cells and control cells

GO ID	GO Description	Corrected *p* value^a^	Cluster frequency^b^	Total frequency^c^
48,513	organ development	9.04E-03	27/107 (10.0%)	1792/17785 (10.0%)
48,731	system development	9.04E-03	32/107 (29.9%)	2422/17785 (13.6%)
42,471	ear morphogenesis	1.97E-02	5/107 (4.6%)	66/17785 (0.3%)
34,754	cellular hormone metabolic process	1.97E-02	5/107 (4.6%)	66/17785 (0.3%)
9913	epidermal cell differentiation	1.97E-02	5/107 (4.6%)	66/17785 (0.3%)
48,856	anatomical structure development	1.97E-02	32/107 (29.9%)	2656/17785 (14.9%)
8285	negative regulation of cell proliferation	2.26E-02	10/107 (9.3%)	379/17785 (2.1%)
90,068	positive regulation of cell cycle process	2.26E-02	4/107 (3.7%)	43/17785 (0.2%)
42,573	retinoic acid metabolic process	2.26E-02	3/107 (2.8%)	17/17785 (0.0%)
9888	tissue development	2.26E-02	14/107 (13.0%)	750/17785 (4.2%)
30,728	ovulation	2.26E-02	3/107 (2.8%)	18/17785 (0.1%)
48,598	embryonic morphogenesis	2.26E-02	9/107 (8.4%)	336/17785 (1.8%)
60,429	epithelium development	2.26E-02	9/107 (8.4%)	337/17785 (1.8%)
48,562	embryonic organ morphogenesis	2.26E-02	6/107 (5.6%)	140/17785 (0.7%)
7166	cell surface receptor linked signaling pathway	2.26E-02	19/107 (17.7%)	1280/17785 (7.1%)
42,904	9-cis-retinoic acid biosynthetic process	2.26E-02	2/107 (1.8%)	4/17785 (0.0%)
42,905	9-cis-retinoic acid metabolic process	2.26E-02	2/107 (1.8%)	4/17785 (0.0%)
35,238	vitamin A biosynthetic process	2.26E-02	2/107 (1.8%)	4/17785 (0.0%)
7275	multicellular organismal development	2.26E-02	33/107 (30.8%)	2971/17785 (16.7%)
6692	prostanoid metabolic process	2.41E-02	3/107 (2.8%)	21/17785 (0.1%)

^a^Corrected *p*-value: After correction, the *p* value in the hypergeometric test

^b^Cluster frequency: the numerator represents the number of each GO term genes and the denominator represents the total number of genes with GO annotation

^c^Total frequency: the numerator represents the number of reference genes annotated in the listed GO term and the denominator represents the number of reference genes with GO annotation

**Fig. 7:**
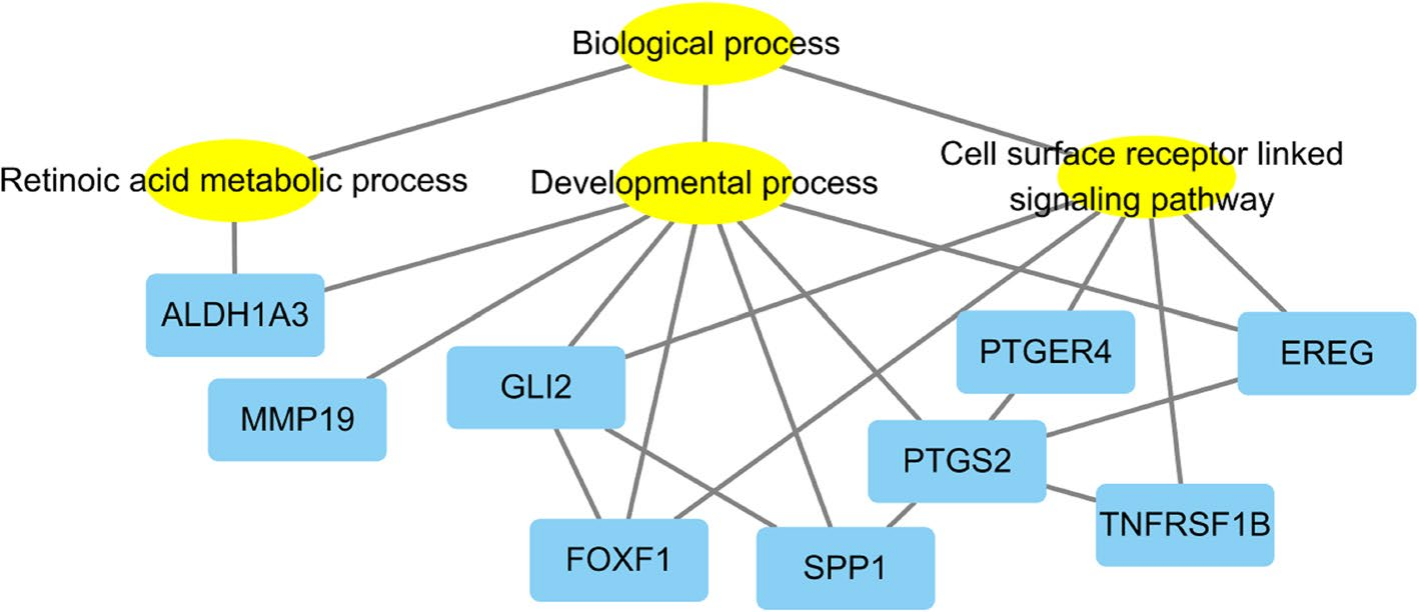
Visualization of gene-gene interaction network and gene ontology (GO) term analysis for selected genes using Cytoscape. Yellow nodes represent GO terms. Blue nodes represent DEGs. Each line represents the interaction between annotated GO terms and DEGs

Additionally, to identify interactions between DEGs involved in these biological processes, we obtained a gene-gene interaction network using the STRING program. Representative genes involved in each process were *EREG*, *FOXF1*, *GLI2*, *PTGS2*, *SPP1*, *MMP19*, *TNFRSF1B*, *PTGER4*, *CLDN18*, and *ALDH1A3*. Fold change values of the DEGs obtained from RNA-seq are shown in Table 3. Relationships between each gene and related GO terms are described in Fig. 7. Analysis of GO enrichment and the gene-gene interaction network showed that many genes activated by CotG-p40 were involved in biological process, including developmental process, signaling, and metabolic process.

**Table 3:** Differential expression of selected genes between CotG-p40 treated cells and control

Gene ID	Gene symbol	Description	Fold change
2069	*EREG*	Epiregulin	3.38
2294	*FOXF1*	Forkhead box F1	7.18
2736	*GLI2*	GLI family zinc finger 2	3.67
5743	*PTGS2*	Prostaglandin-endoperoxide synthase 2	3.20
6696	*SPP1*	Secreted phosphoprotein 1	4.34
4327	*MMP19*	Matrix metallopeptidase 19	4.87
7133	*TNFRSF1B*	TNF receptor superfamily member 1B	5.94
5734	*PTGER4*	Prostaglandin E receptor 4	5.14
51,208	*CLDN18*	Claudin 18	3.46
220	*ALDH1A3*	Aldehyde dehydrogenase 1 family member A3	8.07

### Reverse transcription-quantitative polymerase chain reaction (RT-qPCR) validation

**Fig. 8:**
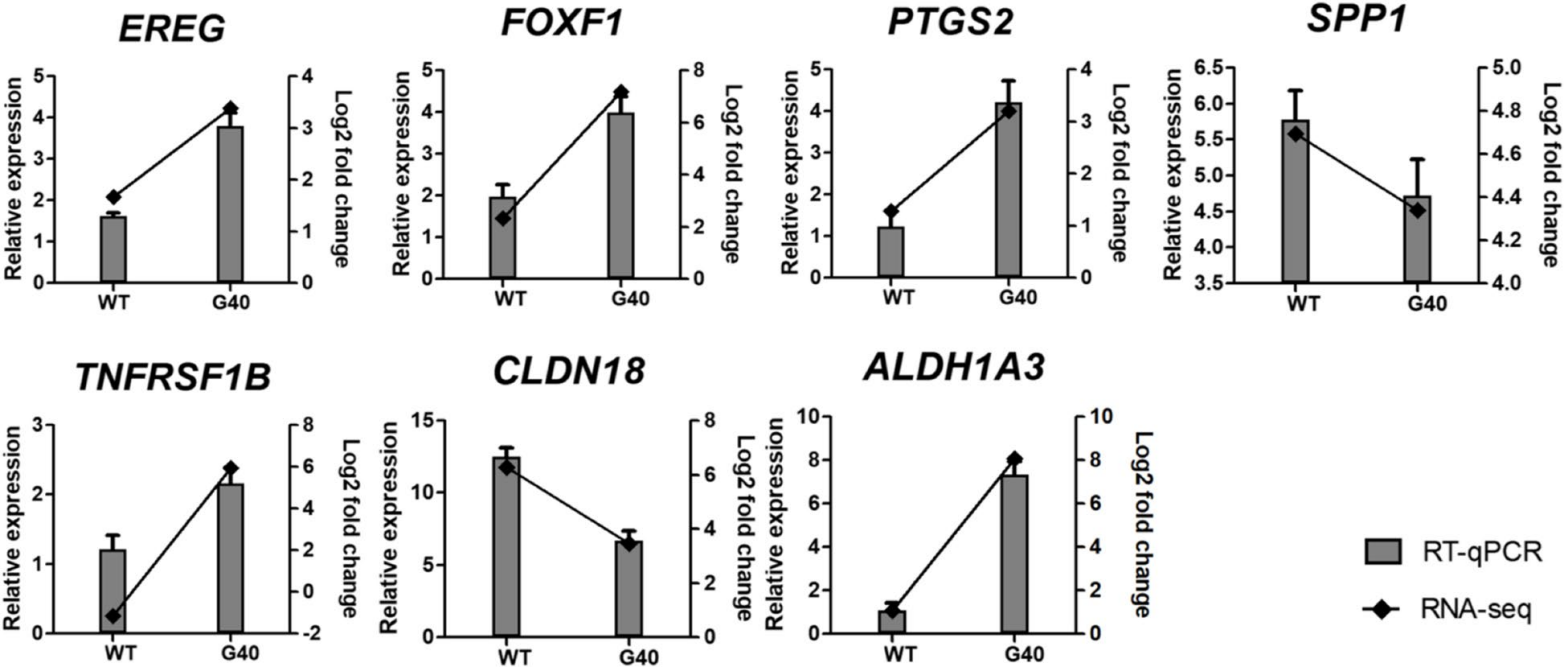
Quantitative reverse transcription PCR (RT-qPCR) analysis data for the seven differentially expressed genes. Experiments were repeated three times. Error bars represent mean ± standard deviation (*n* = 3)

RT-qPCR was performed to validate RNA-seq results. We selected 7 genes (*EREG*, *FOXF1*, *PTGS2*, *SPP1*, *TNFRSF1B*, *CLDN18*, and *ALDH1A3*) associated with probiotic action extracted from gene-gene interaction analysis. Results of comparison between RNA-seq and RT-qPCR are shown in Fig. 8. As a result of RT-qPCR validation, we found that expression levels of all selected genes showed the same tendency as RNA-seq results.

## Discussion

Probiotics help humans maintain homeostasis of internal microbiota and keep the intestine healthy [30]. Soluble proteins secreted by probiotics play key roles in probiotic effects, including alleviation of intestinal diseases such as inflammatory bowel disease (IBD) [31]. p40 is one of soluble proteins secreted by LGG. It controls cell proliferation, apoptosis, and intestinal homeostasis [32]. In our previous study, we have displayed a p75 protein, another LGG derived protein, on the spore surface and verified its stability at various pH and temperature conditions [28].

This study designed CotG-p40 by displaying p40 on the spore surface of *B. subtilis*. RNA-seq was conducted to analyze the transcriptome of CotG-p40 treated HT-29 cells to identify effects of CotG-p40 on human intestinal epithelial cells. The comparison of CotG-p40-stimulated and wild-type spore-stimulated cells to control cells identified 163 and 75 DEGs, respectively (Table S4). This explains that spore surface-displayed p40 mediate transcriptional responses of human intestinal epithelial cells.

A previous study has found that administration of LGG to neonatal mice increases the expression of CLDN3, a tight junction protein, along with the maturation of intestinal barrier function [33]. Another study has also found that LGG mediates CLDN1 to enhance the barrier function of human intestinal epithelial cells [34]. CLDN18, a claudin family protein, also supports mucosal homeostasis of cells [35]. LGG induces the expression of *PTGS2* (also known as *COX2*) in human colon epithelial T84 cells [36]. Morteau et al. have revealed that PTGS2 contributes to maintaining mucosal integrity and healing colitis by preventing colonic injuries from acute mucosal inflammation [37].

p40 transactivates the EGF receptor by activation of matrix metalloproteinases (MMPs) and releases of a metalloproteinase domain-containing protein 17 (ADAM17) mediated heparin binding-epidermal growth factor (HB-EGF) [16, 31]. This suggests that p40 suppresses the apoptosis of the colon and preserves the barrier function [16, 31]. MMP19, a member of the MMP protein family, is involved in maintaining the epithelial barrier of the colon and attenuating colitis development [38]. Likewise, CotG-p40 showed similar effects on intestinal epithelial cells as it up-regulated the expression of *PTGS2, MMP19*, and *CLDN18*. These results indicate that CotG-p40 can help maintain the barrier function of intestinal epithelial cells similar to p40.

Results of GO enrichment analysis and gene-gene interaction network of 163 DEGs from CotG-p40 treated HT-29 cells showed that CotG-p40 was related to biological processes such as developmental process, cell surface receptor linked signaling pathway, and retinoic acid metabolic process. CotG-p40 activated genes including *EREG, ALDH1A3, FOXF1, GLI2, PTGS2, SPP1,* and *MMP19*, are associated with the developmental process. In addition, it stimulated the *ALDH1A3* gene related to the retinoic acid metabolic process and other genes, such as *EREG, FOXF1, GLI2, TNFRSF1B,* and *PTGER4*, associated with cell surface receptor linked signaling pathway.

DEGs related to the developmental process, such as *EREG, SPP1*, and *PTGS2*, are known to be associated with the treatment of IBD. EREG affects the proliferation of intestinal epithelial cells and mediates intestinal wound healing and protection from IBD [39]. Over-expressed SPP1 ameliorates TNF-α induced apoptosis and participates in the mucosal protective mechanism from IBD [40]. PTGS2 is associated with maintaining mucosal integrity and healing of colitis [36]. Up-regulated *EREG*, *SPP1*, and *PTGS2* by CotG-p40 support the claim that it can protect intestinal epithelial cells from intestinal diseases.

*FOXF1* and *GLI2* are genes related to the homeostasis of the intestinal epithelium. The *FOXF1* regulates homeostasis and proliferation of adult intestinal epithelium [41]. GLI2 is a major effector in the hedgehog signaling (Hh) pathway during gut development [42]. The Hh signaling pathway is crucial in that it participates in the development of the gastrointestinal tract and regulates its homeostasis [43]. Therefore, CotG-p40 is expected to help control the homeostasis of intestinal epithelial cells.

*PTGER4* and *TNFRSF1B* belong to cell surface receptor linked signaling pathway. They are associated with wound repair and treatment of intestinal diseases. PTGER4 is one of prostaglandin E2 (PGE_2_) receptors. The expression of PTGER4 can improve wound repair responses of the intestinal epithelium [44]. TNFRSF1B affects wound healing of the IBD by stimulating intestinal cell migration [45].

In this study, *ALDH1A3* annotated both the developmental process and the retinoic acid metabolic process. ALDH1A3 turns retinal into retinoic acid in vitamin A metabolism [46]. Retinoic acid is a biologically active form of vitamin A. It protects the intestinal barrier and determines epithelial integrity [47]. Thus, retinoic acid produced by ALDH1A3 may help maintain the integrity of intestinal epithelial cells.

Considering the effects of wild-type spore on HT-29 cells, wild-type spore treated cells showed higher fold change values of *SPP1* and *CLDN18* than CotG-p40 treated ones. Although the expression level of *SPP1* was only a little higher in wild-type spore treated cells, the expression level of *CLDN18* was about two times higher in wild-type spore treated samples. Rhayat et al. already demonstrated that *B. subtilis* strains reinforce intestinal barrier integrity through up-regulation of the expression of tight junction proteins [48]. Thus, our results are partially consistent with a previous study that have shown probiotic features of *B. subtilis*.

## Conclusions

In conclusion, we displayed p40, a significant protein in probiotic action of LGG, on the surface of *B. subtilis* spore and its effects on transcriptional response of human intestinal epithelial HT-29 cells were evaluated by RNA-seq. There were 10 DEGs acted similarly to the probiotic activity of p40 and LGG. CotG-p40 was associated with the developmental process, cell surface receptor linked signaling pathway, and retinoic acid metabolic process of intestinal epithelial cells. These results suggest that CotG-p40 can regulate proliferation and homeostasis of human intestinal epithelial cells.

## Methods

### Bacterial strains, culture conditions, and transformation

Bacterial strains used in this study are listed in Table 4. *Escherichia coli* DH5α and *B. subtilis* 168 were used for transformation and incubated at 37 °C in Luria-Bertani (LB) medium. *L. rhamnosus* GG ATCC 53103 was used to obtain the *p40* gene and incubated at 37 °C in Man-Rogosa-Sharpe (MRS) medium. *E. coli* DH5α was transformed using the CaCl_2_-mediated method [52]. Transformation of *B. subtilis* was performed according to the method described by Juhas and Ajioka [53]. Ampicillin (50 μg/mL) and kanamycin (50 μg/mL) were used to screen *E. coli* and *B. subtilis* transformants, respectively.

**Table 4:** Bacterial strains, plasmids, and primers used in this study

Bacterial strains, plasmids, or primers	Description	Reference
**Bacterial strains**		
*Escherichia coli* DH5α	*F−, φ 80dlacZ∆M15, ∆(lacZYA-argF)U169, deoR, recA1, endA1, hsdR17(rK−, mK+), phoA, supE44, λ−, thi-1, gyrA96, relA1*	[49]
*Bacillus subtilis* 168	*trpC2*	[50]
*Lacticaseibacillus rhamnosus* GG	ATCC^a^ 53,103	Purchased from ATCC
**Plasmids**		
pUB19	*E. coli*-*B. subtilis* shuttle vector, Ap^r^, Km^r^	[51]
pUB19-*cotG-p40*	Spore display of p40 using the CotG anchor	This study
**Primers**		
CotG-F	5′-CCCTTCG**ACGCGT**^b^CAGCTGGC-3′	This study
CotG-R	5′-ACTTGTGTCGCTTCCTCCTCCTCCTTTGTATTTC TTTTTGACTACCCAGCAATTGCCGTC-3′	This study
p40-F	5′-TACAAAGGAGGAGGAGGAAGCGACACAAGTG CCAGCATCGCATCTAACAAGAGCG-3′	This study
p40-R	5′- AAGGAAAAA**GCGGCCG**^†^CAAAAGGAAAATTA CCGGTGGATGTAAACGTAGCTGCTGGC-3’	This study

^a^ATCC, American type culture collection (Manassas, VA, USA)

^b^Bold letters indicate restriction sites

### Plasmid construction

Plasmids and primers used in this study are shown in Table 4. pUB19 was used as an *E. coli*-*B. subtilis* shuttle vector. The DNA fragment (866 bp) containing *cotG* promoter and structure gene was amplified by PCR using forward primer set cotG-F/cotG-R with chromosomal DNA of *B. subtilis*. The DNA frangment (1206 bp) containing *p40* structure gene except 84 bp of the signal peptide sequence was amplified from LGG chromosome using primer set p40-F/p40-R. These amplified *cotG* and *p40* genes were fused by overlap extension PCR using primer set p40-F /cotG-R. To provide flexibility and mobility between functional domains, a flexible linker (Gly-Gly-Gly-Gly-Ser) was inserted between *cotG* and *p40* genes [54]. The amplified *cotG-p40* fragment (2042 bp) was digested with restriction enzymes *Not*I and *Mlu*I and ligated into pUB19 shuttle vector digested with the same enzymes.

### Preparation of spores

*B. subtilis* 168 containing pUB19*-cotG-p40* was incubated at 37 °C for 62 h in shaking incubator at 150 rpm in Difco Sporulation Medium (DSM) consisting of the 0.8% nutrient broth, 0.025% MgSO_4_‧7H_2_O, 0.1% KCl, 10 M MnCl_2_, 1 M FeSO_4_‧7H_2_O, 1 M Ca (NO_3_)_2_. After cultivation, the spores and the sporangial cells of *B. subtilis* 168 recombinant plasmid were collected by centrifugation at 5000×*g* for 10 min at 4 °C. Spores were obtained with a previously reported method [55]. Shortly, these spores were resuspended in 50 mM sodium phosphate buffer (pH 7.2) and treated with lysozyme to destroy residual sporangial cells at 4 °C for 1 h. After centrifugation at 5000×*g* for 10 min at 4 °C, the purified spores were washed with 1 M NaCl and 1 M KCl. After the final washing with 50 mM sodium phosphate buffer, spores were resuspended in sterile phosphate-buffered saline (PBS, pH 7.4) and stored at 4 °C. Plating of serial dilutions was carried out on LB agar plates to count spores. The final concentration of spores was adjusted to 10^5^ spores/mL.

### Preparation of peptidoglycan

The peptidoglycan extraction was carried out based on the method as described Atrih et al. [56]. Briefly, the bacterial pellet recovered from a 250 mL of the mid-log phase cell culture of *B. subtilis*. The pellet was boiled, and then centrifuged at 14,000×*g* for 8 min at 4 °C. The collected pellet was resuspended in boiling 5% (W/V) sodium dodecyl sulfate (SDS) and incubated for 25 min. After centrifugation, the pellet was resuspended in boiling 4% (W/V) SDS and incubated for 15 min. The insoluble residue was collected and washed in distilled water (DW) 6–7 times to eliminate SDS. Then the sediment was treated with proteinase K (2 mg/mL) and trypsin (200 μg/mL) for 1 h and 16 h, separately, at 37 °C to remove covalently attached proteins. Then the insoluble materials were collected by centrifuge and incubated in 48% (V/V) hydrofluoric acid for 24 h at 4 °C. After centrifugation, the pH of the insoluble cell wall was adjusted to 7.0 by resuspending in Tris-HCl buffer (50 mM, pH 7) and washing five times with cold DW. Finally, the PG extract was suspended in 1.5 mL DW and stored at 4 °C for further analysis.

### PGH activity assay of CotG-p40

The PGH activity of CotG-p40 was measured using the ninhydrin method [57]. The PG of *B. subtilis* was used as a substrate. Briefly, 30 μL of CotG-p40 at different concentrations (1.4, 2.8, 4.2, 5.6, and 7.0 × 10^3^ spores/mL) was mixed with 30 μL of PG extract. The mixtures were incubated at 37 °C for 15 min. After the reaction, the unhydrolyzed PG extract was precipitated by centrifugation at 14,000×*g* for 5 min. Then, 30 μL of the supernatant was taken in a new tube and 3 μL of 2% (W/V) ninhydrin solution was added. The reaction solution was incubated in boiling water bath for 5 min and cooled to room temperature for 5 min in ice bath. The absorbance of the reaction mixture was measured at 570 nm using a Nanodrop 2000 spectrophotometer (Thermo Fisher Scientific, Wilmington, DE, USA). PG treated with DW instead of CotG-p40 was used as a blank. PG treated with wild-type spores was used as a control.

### Stability test

The thermal and pH stability of CotG-p40 was determined under various temperature and pH conditions based on ninhydrin assay. The thermal stability of CotG-p40 was examined by incubating 30 μL of spores (7.0 × 10^3^ spores/mL) at various temperatures (40, 50, 60, 70, and 80 °C) for 15 min and cooling to room temperature (25 °C). The pH stability of CotG-p40 was determined by incubating 30 μL of spores in various pH buffers ranging from 2 to 8 for 15 min. Then all samples were washed at least three times with PBS. After each temperature and pH treatment, all samples were incubated with the PG extract at 37 °C for 15 min. The residual activity of each sample was measured with the ninhydrin assay as already described. Relative activity was calculated by defining the respective original activity measured at 25 °C and pH 7 as 100%.

### Cell culture and spore treatment

Human intestinal epithelial cell line HT-29 was cultured in Dulbecco’s modified Eagle medium (DMEM) supplemented with 10% heat-inactivated fetal bovine serum (FBS), 100 U/mL penicillin, and 100 μg/mL streptomycin. Cells were incubated at 37 °C in a 5% CO_2_ incubator. The culture medium was changed every other day. HT-29 cells were then seeded into 12-well plates at a density of 4 × 10^5^ cells/mL. After cells reached appropriate confluence, the medium was removed and cells were treated with spores (10^5^ spores/mL) for 3 h. Cells treated with PBS instead of spores were used as a control.

### RNA extraction, library construction, and RNA-sequencing

Total RNA was extracted using TRIzol reagent (Invitrogen, Waltham, MA, USA) according to the manufacturer’s instruction. Total RNA extraction from each sample was performed independently in triplicate. Total RNA samples were treated with RNase-free DNaseI to eliminate possible DNA contaminants. The quality and quantity of the RNA were verified by a NanoDrop 2000 UV spectrophotometer (Thermo Scientific, Waltham, MA, USA). Only RNA samples meeting the quality control parameters were used for RNA-seq and qRT-PCR.

Libraries were constructed using a TruSeq Stranded mRNA LT sample prep kit (Illumina, San Diego, CA, USA) following the protocol outlined by Illumina (https://support.illumina.com/sequencing/sequencing_kits/truseq-stranded-mrna.html). Briefly, mRNA was purified and fragmented from 1 μg of total RNA using oligo dT magnetic beads. The fragmented mRNAs were synthesized as single-stranded cDNAs using random hexamer primers. Using this as a template, double-stranded cDNA was obtained. Subsequently, end repair, A-tailing, and adapter ligation were performed. Then, the products were amplified with PCR to generate the final cDNA library.

RNA sequencing was performed as paired-end (2 × 101 bp) on an Illumina NovaSeq 6000 platform (Illumina, San Diego, CA, USA). Sequencing quality control was carried out using FastQC v0.11.7 (http://www.bioinformatics.babraham.ac.uk/projects/fastqc/). To obtain clean reads, adapter and low-quality reads from raw data were removed using a Trimmomatic 0.38 [58]. Clean reads were mapped to human reference genome (GRCh37) using HISAT2 version 2.1.0 and Bowtie2 2.3.4.1 software. The sequence data from this study have been deposited into Sequence Read Archive (http://www.ncbi.nlm.nih.gov/sra) under accession number PRJNA746714.

### Identification of differentially expressed genes (DEGs)

The mapped reads for each sample were assembled using StringTie (https://ccb.jhu.edu/software/stringtie/). StringTie and DESeq2 were used to estimate the expression levels of transcripts [59]. The value of fragments per kilobase of transcript per million mapped reads (FPKM) was used to normalize gene expression level. Genes meeting the criteria of |fold change| > 2 and raw *p*-value < 0.05 were defined as DEGs.

### Gene ontology (GO) enrichment analysis and gene-gene interaction network analysis

To identify biological functions of DEGs, Biological Network Gene Ontology (BiNGO) tool was used to perform GO enrichment analysis [60]. The *p*-values were adjusted by Benjamini-Hochberg correction for multiple hypothesis testing. The GO terms with a corrected *p*-value < 0.05 were considered significantly enriched. To identify interactions between DEGs, gene-gene interaction network was constructed using Search Tool for the Retrieval of Interacting Genes (STRING, https://string-db.org/) with a minimum required interaction score set at > 0.4 [61]. GO enrichment analysis and gene-gene interaction network analysis were visualized using Cytoscape v3.7.2 [62].

### Quantitative real-time PCR (qRT-PCR) validation

The seven genes (*EREG*, *FOXF1*, *PTGS2*, *SPP1*, *TNFRSF1B*, *CLDN18*, and *ALDH1A3*) associated with probiotic action were selected and validated by RT-qPCR. cDNA was synthesized from total RNA using random hexamers (Roche, Basel, Switzerland) and M-MLV reverse transcriptase (Promega, Madison, WI, USA). RT-qPCR was performed on a CFX Connect™ Real-Time System (Bio-rad, Hercules, CA, USA) under the conditions at 95 °C for 10 s, followed by 40 cycles of 95 °C for 5 s and 60 °C for 31 s using SYBR-Green PCR Master Mix kit (Takara, Shiga, Japan). Primer sets were listed in Table 5. *GAPDH* gene was used as an internal control to normalize data. Relative gene expression levels were calculated using a comparative cycle threshold method [63].

**Table 5:** Primer sequences used for RT-qPCR

Gene symbol	Forward primer (5′ → 3′)	Reverse primer (5′ → 3′)	Accession number
*GAPDH*	AAGGTGAAGGTCGGAGTCAA	ATGACAAGCTTCCCGTTCTC	NM_002046
*EREG*	CTTATCACAGTCGTCGGTTCCAC	GCCATTCAGACTTGCGGCAACT	NM_001432
*FOXF1*	AGCAGCCGTATCTGCACCAGAA	CTCCTTTCGGTCACACATGCTG	NM_001451
*PTGS2*	CGGTGAAACTCTGGCTAGACAG	GCAAACCGTAGATGCTCAGGGA	NM_000963
*SPP1*	CGAGGTGATAGTGTGGTTTATGG	GCACCATTCAACTCCTCGCTTTC	NM_000582
*TNFRSF1B*	CGTTCTCCAACACGACTTCATCC	ACGTGCAGACTGCATCCATGCT	NM_001066
*CLDN18*	ATGGAGGACTCTGCCAAAGCCA	TGGACATCCAGAAGTTAGTCACC	NM_016369
*ALDH1A3*	CTGCTACAACGCCCTCTATGCA	GTCGCCAAGTTTGATGGTGACAG	NM_000693

### Statistical analysis

For RT-qPCR data, all experiments were performed in triplicates. All the numeric values were expressed as mean ± standard deviation (SD). All statistical analyses were carried out using unpaired *t*-test of GraphPad Prism 5.0 software (GraphPad, San Diego, CA, USA).

## Supplementary Information

**Additional file 1: Table S1.** Quality control and mapping of raw reads. **Table S2.** All differentially expressed genes (DEGs) between CotG-p40 treated HT-29 cells and control. **Table S3.** All differentially expressed genes (DEGs) between CotG-p40-treated and wild-type spore-treated HT-29 cells. **Table S4.** All differentially expressed genes (DEGs) between wild-type spore treated HT-29 cells and control. **Table S5.** Gene ontology (GO) enrichment analysis of 163 differentially expressed genes (DEGs) between CotG-p40-treated HT-29 cells and control. **Table S6.** Gene ontology (GO) enrichment analysis of 147 differentially expressed genes (DEGs) between CotG-p40- and wild-type spore-treated HT-29 cells. **Table S7.** Gene ontology (GO) enrichment analysis of 147 differentially expressed genes (DEGs) between wild-type-spore treated HT-29 cells and control. **Fig. S1.** Determination of the peptidoglycan hydrolase activity of wild-type spore. After treatment of peptidoglycan with different concentrations of wild-type spore at 37°C for 15 min, the absorbance of each sample was measured at 570 nm. All tests were performed in triplicate, and the data are presented as mean ± standard deviation. **Fig. S2.** Gene ontology (GO) analysis for DEGs between CotG-p40- and wild-type spore-treated HT-29 cells. **Fig. S3.** Gene ontology (GO) analysis for DEGs between wild-type spore-treated HT-29 cells and control.

## Data Availability

The datasets supporting the conclusions of this article are included within the article and its additional files. The datasets presented in this study are available in NCBI Sequence Read Archive (SRA) (http://www.ncbi.nlm.nih.gov/sra) with the accession number PRJNA746714.

## Abbreviations

ADAM17: A metalloproteinase domain-containing protein 17
BiNGO: Biological network gene ontology
DEG: Differentially expressed gene
DMEM: Dulbecco’s modified Eagle medium
DSM: Difco sporulation medium
EGF: Epidermal growth factor
FBS: Fetal bovine serum
FPKM: Fragments per kilobase of transcript per million mapped reads
GO: Gene ontology
HB-EGF: Heparin binding-epidermal growth factor
Hh signaling: Hedgehog signaling
IBD: Inflammatory bowel disease
LB: Luria-Bertani
LGG: *Lacticaseibacillus rhamnosus* GG
MMPs: Matrix metalloproteinases
MRS: Man-Rogosa-Sharpe
PBS: Phosphate-buffered saline
PCR: Polymerase chain reaction
PGE_2_: Prostaglandin E2
qRT-PCR: Quantitative real-time PCR
RNA-seq: RNA-sequencing
SD: Standard deviation
STRING: Search tool for the retrieval of interacting genes
