# Supplementary material for: Transcriptional responses of human intestinal epithelial HT-29 cells to spore-displayed p40 derived from Lacticaseibacillus rhamnosus GG
Source: BMC Microbiol. 2022 Dec 22;22:316. doi: 10.1186/s12866-022-02735-3 (PMC9772600; doi:10.1186/s12866-022-02735-3)
Supplement: Supplementary file 1 — Additional file 1: Table S1. Quality control and mapping of raw reads. Table S2. All differentially expressed genes (DEGs) between CotG-p40 treated HT-29 cells and control. Table S3. All differentially expressed genes (DEGs) between CotG-p40-treated and wild-type spore-treated HT-29 cells. Table S4. All differentially expressed genes (DEGs) between wild-type spore treated HT-29 cells and control. Table S5. Gene ontology (GO) enrichment analysis of 163 differentially expressed genes (DEGs) between CotG-p40-treated HT-29 cells and control. Table S6. Gene ontology (GO) enrichment analysis of 147 differentially expressed genes (DEGs) between CotG-p40- and wild-type spore-treated HT-29 cells. Table S7. Gene ontology (GO) enrichment analysis of 147 differentially expressed genes (DEGs) between wild-type-spore treated HT-29 cells and control. Fig. S1. Determination of the peptidoglycan hydrolase activity of wild-type spore. After treatment of peptidoglycan with different concentrations of wild-type spore at 37°C for 15 min, the absorbance of each sample was measured at 570 nm. All tests were performed in triplicate, and the data are presented as mean ± standard deviation. Fig. S2. Gene ontology (GO) analysis for DEGs between CotG-p40- and wild-type spore-treated HT-29 cells. Fig. S3. Gene ontology (GO) analysis for DEGs between wild-type spore-treated HT-29 cells and control. [file 12866_2022_2735_MOESM1_ESM.pdf]

Journal name:

BMC Microbiology

Manuscript title:

Transcriptional responses of human intestinal epithelial HT-29 cells to spore-displayed p40 derived from *Lacticaseibacillus rhamnosus* GG

The names of the authors:

Soo Ji Kang[1,*], Jeong A Moon[1,*], Do Yeong Son[1], and Kwang Won Hong[1, †]

* These two authors contributed equally to this work.

The affiliation and address of the authors:

[1] Department of Food Science and Biotechnology, College of Life Science and Biotechnology, Dongguk University, Goyang-si 10326, Republic of Korea

[†] **Corresponding author:**

Kwang Won Hong (E-mail address: hkwon@dongguk.edu, Tel: +82-31-961-5140, Fax: +82-31-961-5108)

Table S1 Quality control and mapping of raw reads

Sample[a]	Raw data		Clean data		Mapped reads
	Raw reads	Q30[b] (%)	Clean reads	Q30 (%)	
CotG-p40	63,029,378	95.20	62,200,250 (98.68%)	95.85	60,914,837 (97.93%)
wild type spore	81,130,202	95.32	80,150,244 (98.79%)	95.92	79,191,914 (98.8%)
control	78,305,704	95.38	77,090,466 (98.45%)	96.08	76,123,734 (98.75%)

[a] Each sample represents HT-29 cells unstimulated or stimulated with wild-type spore or CotG-p75

[b] Q30 indicates the percentage of sequencing data with an error rate less than 0.1%

Table S2 All differentially expressed genes (DEGs) between CotG-p40 treated HT-29 cells and control

Gene_ID	Transcript_ID	Gene_symbol	Description	Fold change	*p*-value
220	NM_000693, NM_001293815	ALDH1A3	aldehyde dehydrogenase 1 family member A3	8.070459	0.000788023
604	NM_001130845, NM_001134738, NM_001706	BCL6	BCL6 transcription repressor	-3.865522	0.024490376
868	NM_001321786, NM_001321788, NM_001321789, NM_001321790, NM_001321791, NM_001321793, NM_001321794, NM_001321795, NM_001321796, NM_001321797, NM_001321798, NM_001321799, NM_001321806, NM_001321807, NM_001321808, NM_001321811, NM_001321813, NM_001321816, NM_001321820, NM_001321822, NM_170662, NR_135806, NR_135807, NR_135808, NR_135809, NR_135810, NR_135811, NR_135812	CBLB	Cbl proto-oncogene B	-3.365272	0.042692413
1018	NM_001258	CDK3	cyclin dependent kinase 3	31.464692	0.000104849
1026	NM_000389, NM_001220777, NM_001220778, NM_001291549, NM_078467	CDKN1A	cyclin dependent kinase inhibitor 1A	3.366602	0.041154263
1543	NM_000499, NM_001319216, NM_001319217	CYP1A1	cytochrome P450 family 1 subfamily A member 1	380.253103	1.27878E-13
1545	NM_000104	CYP1B1	cytochrome P450 family 1 subfamily B member 1	11.600687	0.000117657
1906	NM_001168319, NM_001955	EDN1	endothelin 1	-3.718983	0.028360398
2069	NM_001432	EREG	epiregulin	3.384286	0.040108184
2199	NM_001004019, NM_001165035, NM_001998	FBLN2	fibulin 2	-10.335995	0.007434817
2294	NM_001451	FOXF1	forkhead box F1	7.175940	0.03224147
2672	NM_001127215, NM_001127216, NM_005263	GFI1	growth factor independent 1 transcriptional repressor	-4.312705	0.049814168
2702	NM_005266, NM_181703	GJA5	gap junction protein alpha 5	3.844038	0.025965012

2736	NM_001371271, NM_005270	GLI2	GLI family zinc finger 2	3.672842	0.035113585
2840	NM_001161415, NM_001161416, NM_001161417, NM_005291	GPR17	G protein-coupled receptor 17	7.285001	0.004543817
2886	NM_001030002, NM_001242442, NM_001242443, NM_001330207, NM_005310	GRB7	growth factor receptor bound protein 7	-5.556027	0.005064973
3009	NM_005322	H1-5	H1.5 linker histone, cluster member	4.369575	0.040683222
3038	NM_001199280, NM_005329, NM_138612	HAS3	hyaluronan synthase 3	4.493599	0.016121682
3268	NM_006076	AGFG2	ArfGAP with FG repeats 2	-3.245761	0.048261627
3283	NM_000862, NM_001328615	HSD3B1	hydroxy-delta-5-steroid dehydrogenase, 3 beta- and steroid delta-isomerase 1	3.238738	0.049903713
3887	NM_002281	KRT81	keratin 81	4.374395	0.046781564
4327	NM_001272101, NM_002429, NR_073606	MMP19	matrix metallopeptidase 19	4.866968	0.042028125
4345	NM_001004196, NM_001318826, NM_001318828, NM_001318830, NM_001365851, NM_001365852, NM_001365853, NM_001365854, NM_001365855, NM_005944, NR_158642	CD200	CD200 molecule	-9.024298	0.006591493
4487	NM_002448	MSX1	msh homeobox 1	5.087003	0.029884417
4602	NM_001130172, NM_001130173, NM_001161656, NM_001161657, NM_001161658, NM_001161659, NM_001161660, NM_005375, NR_134958, NR_134959, NR_134960, NR_134961, NR_134962, NR_134963, NR_134964, NR_134965	MYB	MYB proto-oncogene, transcription factor	-4.774353	0.010201443
4886	NM_000909	NPY1R	neuropeptide Y receptor Y1	-4.375857	0.038568237
5013	NM_001199770, NM_014562, NR_130153	OTX1	orthodenticle homeobox 1	-4.142165	0.020449066
5166	NM_002612	PDK4	pyruvate dehydrogenase kinase 4	-4.028450	0.023307682

5284	NM_002644	PIGR	polymeric immunoglobulin receptor	-4.673274	0.032224685
5582	NM_001316329, NM_002739	PRKCG	protein kinase C gamma	5.025311	0.009181464
5734	NM_000958	PTGER4	prostaglandin E receptor 4	5.141547	0.03850259
5743	NM_000963	PTGS2	prostaglandin-endoperoxide synthase 2	3.199421	0.049766489
5801	NM_001207015, NM_001207016, NM_002849, NM_130846, NR_073474	PTPRR	protein tyrosine phosphatase receptor type R	-4.752584	0.013903006
6364	NM_001130046, NM_004591	CCL20	C-C motif chemokine ligand 20	5.137131	0.010981431
6398	NM_003004	SECTM1	secreted and transmembrane 1	5.991203	0.004952333
6402	NM_000655, NR_029467	SELL	selectin L	-5.213550	0.036672434
6696	NM_000582, NM_001040058, NM_001040060, NM_001251829, NM_001251830	SPP1	secreted phosphoprotein 1	4.340423	0.015205242
6698	NM_001199828, NM_005987	SPRR1A	small proline rich protein 1A	7.161585	0.007458047
6699	NM_003125	SPRR1B	small proline rich protein 1B	5.154781	0.009103057
6707	NM_001097589, NM_005416	SPRR3	small proline rich protein 3	4.188963	0.019846355
7049	NM_001195683, NM_001195684, NM_003243, NR_036634	TGFBR3	transforming growth factor beta receptor 3	-3.421881	0.040398838
7128	NM_001270507, NM_001270508, NM_006290	TNFAIP3	TNF alpha induced protein 3	3.630113	0.033869628
7133	NM_001066	TNFRSF1B	TNF receptor superfamily member 1B	5.941573	0.005769765
7433	NM_001251882, NM_001251883, NM_001251884, NM_001251885, NM_004624	VIPR1	vasoactive intestinal peptide receptor 1	3.585748	0.032043242
7832	NM_006763	BTG2	BTG anti-proliferation factor 2	-3.662242	0.030344258
8000	NM_005672, NR_033343	PSCA	prostate stem cell antigen	-5.952234	0.004193956

8120	NM_001278511, NM_001278512, NM_001348440, NM_001348441, NM_004644	AP3B2	adaptor related protein complex 3 subunit beta 2	9.795917	0.001550881
8482	NM_001146029, NM_001146030, NM_003612	SEMA7A	semaphorin 7A (John Milton Hagen blood group)	7.401501	0.001427483
8743	NM_001190942, NM_001190943, NM_003810, NR_033994	TNFSF10	TNF superfamily member 10	-3.623458	0.0340518
9536	NM_004878	PTGES	prostaglandin E synthase	5.562408	0.006145148
9586	NM_001011666, NM_004904, NM_182898, NM_182899	CREB5	cAMP responsive element binding protein 5	4.010641	0.023751501
9788	NM_001282971, NM_001282974, NM_001363294, NM_001363295, NM_001363296, NM_001363297, NM_001363298, NM_001363299, NM_001363300, NM_001363301, NM_001363302, NM_014751	MTSS1	MTSS I-BAR domain containing 1	4.768422	0.010013896
9940	NM_001321153, NM_007335, NM_007337	DLEC1	DLEC1 cilia and flagella associated protein	3.521017	0.042185006
10008	NM_005472	KCNE3	potassium voltage-gated channel subfamily E regulatory subunit 3	-3.497363	0.03586849
10023	NM_005479	FRAT1	FRAT regulator of WNT signaling pathway 1	-3.795971	0.036076258
10170	NM_001142270, NM_001142271, NM_001289763, NM_199204	DHRS9	dehydrogenase/reductase 9	5.673434	0.004941883
10194	NM_001308210, NM_005786	TSHZ1	teashirt zinc finger homeobox 1	-3.858808	0.024768288
10957	NM_006813	PNRC1	proline rich nuclear receptor coactivator 1	-3.566566	0.035663935
23024	NM_001303139, NM_001303140, NM_001303141, NM_001303142, NM_015009	PDZRN3	PDZ domain containing ring finger 3	-5.168969	0.020964854
23254	NM_001017999, NM_001018000, NM_001018001, NM_001370229, NM_001370230, NM_001370231, NM_015209, NM_201628	KAZN	kazrin, periplakin interacting protein	3.670838	0.04285701
23285	NM_015237	KIAA1107	KIAA1107	-7.074211	0.003899931

25758	NM_012194	KIAA1549L	KIAA1549 like	6.740014	0.008233828
26959	NM_001244262, NM_012257	HBP1	HMG-box transcription factor 1	-4.433073	0.013812703
27113	NM_001127240, NM_001127241, NM_001127242, NM_014417	BBC3	BCL2 binding component 3	-4.284813	0.016398991
29114	NM_001008272, NM_001008273, NM_013259	TAGLN3	transgelin 3	16.088913	9.53738E-05
51208	NM_001002026, NM_016369	CLDN18	claudin 18	3.456191	0.040149587
51676	NM_001202429, NM_016150	ASB2	ankyrin repeat and SOCS box containing 2	4.458984	0.034222603
51702	NM_016233	PADI3	peptidyl arginine deiminase 3	6.101730	0.004338837
54567	NM_019074	DLL4	delta like canonical Notch ligand 4	-3.584589	0.044084393
54800	NM_001349413, NM_001349414, NM_001349415, NM_001349416, NM_001349417, NM_001349418, NM_001349419, NM_001349420, NM_001349421, NM_001349422, NM_001349423, NM_001349424, NM_001349425, NM_001349426, NM_001349428, NM_001349429, NM_017644, NR_146169, NR_146170	KLHL24	kelch like family member 24	-5.187375	0.007443443
54866	NM_001130143, NM_017726	PPP1R14D	protein phosphatase 1 regulatory inhibitor subunit 14D	-3.602607	0.048872231
56204	NM_001286495, NM_019600, NR_104457	FAM214A	family with sequence similarity 214 member A	-3.401726	0.04196421
57214	NM_001293298, NM_001293304, NM_018689	CEMIP	cell migration inducing hyaluronidase 1	3.460785	0.036932353
57713	NM_001018039, NM_001029880	SFMBT2	Scm like with four mbt domains 2	6.560968	0.013339641
63982	NM_001313726, NM_001313727, NM_031418	ANO3	anoctamin 3	-4.173597	0.026688556
64108	NM_022147	RTP4	receptor transporter protein 4	-7.459568	0.014252316
64838	NM_022823	FNDC4	fibronectin type III domain containing 4	4.753677	0.034864293
78990	NM_023112	OTUB2	OTU deubiquitinase, ubiquitin aldehyde binding 2	4.281691	0.017134801
79094	NM_001142776, NM_024111	CHAC1	ChaC glutathione specific gamma-glutamylcyclotransferase 1	3.903845	0.023371687

79170	NM_024320	PRR15L	proline rich 15 like	-3.614406	0.032076612
79413	NM_024508	ZBED2	zinc finger BED-type containing 2	4.946791	0.01583439
79661	NM_001256552, NM_001352519, NM_001352520, NM_024608, NR_046311	NEIL1	nei like DNA glycosylase 1	7.109117	0.003632326
79966	NM_001037582, NM_024906	SCD5	stearoyl-CoA desaturase 5	4.418635	0.025343964
80318	NM_001135953, NM_025211	GKAP1	G kinase anchoring protein 1	-3.957812	0.034771066
80352	NM_025236, NM_170769	RNF39	ring finger protein 39	-3.391919	0.043302111
83719	NM_001145524, NM_031477	YPEL3	yippee like 3	-4.316764	0.016624475
83992	NM_001363349, NM_001363350, NM_001363351, NM_033427	CTTNBP2	cortactin binding protein 2	-4.334657	0.020921183
84129	NM_032169, NR_132426, NR_132427, NR_132428	ACAD11	acyl-CoA dehydrogenase family member 11	5.933175	0.005133003
84159	NM_001244638, NM_032199	ARID5B	AT-rich interaction domain 5B	-4.910996	0.008703441
84189	NM_032229	SLITRK6	SLIT and NTRK like family member 6	-4.481786	0.013103078
84623	NM_001161707, NM_001301097, NM_032531	KIRREL3	kirre like nephrin family adhesion molecule 3	4.395316	0.026126751
84666	NM_032579	RETNLB	resistin like beta	-4.898899	0.046781564
84992	NM_001042616	PIGY	phosphatidylinositol glycan anchor biosynthesis class Y	4.083029	0.021795188
85344	gene-KRT89P	KRT89P	keratin 89 pseudogene	5.742496	0.027120138
85409	NM_001271082, NM_033120	NKD2	NKD inhibitor of WNT signaling pathway 2	3.623652	0.031411636
85442	NM_001347864, NM_001347865, NM_001347866, NM_152643	KNDC1	kinase non-catalytic C-lobe domain containing 1	-7.068386	0.018080005
85495	NR_002312	RPPH1	ribonuclease P RNA component H1	5.645883	0.013852865
90853	NM_001281987, NM_001281988, NM_144569	SPOCD1	SPOC domain containing 1	8.980969	0.003598289
121268	NM_001303126, NM_144593, NR_130123	RHEBL1	RHEB like 1	-4.151049	0.029325497

130574	NM_001195685, NM_194317	LYPD6	LY6/PLAUR domain containing 6	-5.136673	0.015398876
140893	NM_080833	RBBP8NL	RBBP8 N-terminal like	-3.683656	0.031019454
147011	NM_001304949, NM_001304951, NM_001304952, NM_001304953, NM_001304954, NM_001366301, NM_001366302, NM_001366303, NM_152465	PROCA1	protein interacting with cyclin A1	-8.241933	0.009072677
147111	NM_178493	NOTUM	notum, palmitoleoyl-protein carboxylesterase	4.374395	0.046781564
148709	NR_002929	LOC148709	actin gamma 1 pseudogene	-9.193415	0.010510413
151050	NM_001307976, NM_152519	KANSL1L	KAT8 regulatory NSL complex subunit 1 like	-3.646902	0.031508854
164312	NM_152611	LRRN4	leucine rich repeat neuronal 4	5.492264	0.006360969
171523	NR_144551	CYP2T1P	cytochrome P450 family 2 subfamily T member 1, pseudogene	-4.772413	0.029505815
219855	NM_001145290, NM_198277	SLC37A2	solute carrier family 37 member 2	8.688348	0.000693247
221472	NM_173558	FGD2	FYVE, RhoGEF and PH domain containing 2	8.996137	0.002140559
222901	NR_026673	RPL23P8	ribosomal protein L23 pseudogene 8	-4.276718	0.04226773
245973	NM_001039362, NM_144583	ATP6V1C2	ATPase H+ transporting V1 subunit C2	4.358624	0.021867255
254439	NM_001136485, NM_001353554	C11orf86	chromosome 11 open reading frame 86	4.477463	0.023066449
255488	NM_182757	RNF144B	ring finger protein 144B	-4.037730	0.023045161
266727	NM_153487	MDGA1	MAM domain containing glycosylphosphatidylinositol anchor 1	10.531427	0.015150304
267004	NM_170753	PGBD3	piggyBac transposable element derived 3	20.873326	0.004429481
283876	NR_033904	LINC00921	long intergenic non-protein coding RNA 921	-4.425784	0.044985511
285154	NR_027252	CYP1B1-AS1	CYP1B1 antisense RNA 1	4.235279	0.022545147
286204	NM_173689, NR_104603	CRB2	crumbs cell polarity complex component 2	-5.488553	0.010718026
326321	gene-RPS24P2	RPS24P2	ribosomal protein S24 pseudogene 2	-6.908255	0.033664757
338817	NR_033890	LINC01252	long intergenic non-protein coding RNA 1252	-5.149966	0.031346675

339894	NR_034007	LINC00880	long intergenic non-protein coding RNA 880	4.656501	0.017024419
374907	NM_198540	B3GNT8	UDP-GlcNAc:betaGal beta-1,3-N-acetylglucosaminyltransferase 8	-4.049818	0.034690189
375791	NM_199001	CYSRT1	cysteine rich tail 1	5.106239	0.008410819
386593	NR_027928	CHKB-CPT1B	CHKB-CPT1B readthrough (NMD candidate)	-6.348142	0.011397017
388403	NM_001005404	YPEL2	yippee like 2	-3.532007	0.036335813
389058	NM_001003845	SP5	Sp5 transcription factor	-5.130278	0.009955665
401251	NM_001039651	SAPCD1	suppressor APC domain containing 1	-4.623634	0.043342751
440925	NR_027433	LINC01124	long intergenic non-protein coding RNA 1124	-4.538864	0.040683222
474338	NR_002190	SUMO1P3	SUMO1 pseudogene 3	4.331386	0.026298266
493861	NM_001008394	EID3	EP300 interacting inhibitor of differentiation 3	3.967700	0.039579885
619349	gene-SETP3	SETP3	SET pseudogene 3	5.442022	0.03224147
643932	gene-RPS3AP20	RPS3AP20	ribosomal protein S3a pseudogene 20	-6.497146	0.019434951
723788	NR_148965	MIG7	mig-7	4.624968	0.049219036
729291	NR_148936	LOC729291	uncharacterized LOC729291	-6.073921	0.00718397
100128640	NR_028389	ACVR2B-AS1	ACVR2B antisense RNA 1	-12.621155	0.002906428
100129484	NM_001354886	LOC100129484	uncharacterized LOC100129484	-3.602607	0.048872231
100131378	NM_001166692	C11orf91	chromosome 11 open reading frame 91	6.274630	0.010496335
100131956	gene-RPS3P5	RPS3P5	ribosomal protein S3 pseudogene 5	14.320182	0.020717199
100151684	NR_023344	RNU6ATAC	RNA, U6atac small nuclear (U12-dependent splicing)	-10.229753	0.026706022
100271603	gene-RPL13AP24	RPL13AP24	ribosomal protein L13a pseudogene 24	-5.807880	0.018852638
100287898	NM_001242672	TTC34	tetratricopeptide repeat domain 34	5.929476	0.011639558
100420326	gene-LOC100420326	LOC100420326	ferritin heavy chain like 17 pseudogene	-18.912176	0.002525671
100422595	gene-PCMTD1P3	PCMTD1P3	protein-L-isoaspartate (D-aspartate) O-methyltransferase domain containing 1 pseudogene 3	-10.229753	0.026706022

100506810	NR_038856	LINC01132	long intergenic non-protein coding RNA 1132	-3.564224	0.047643114
100526760	NM_001316331	ABHD14A-ACY1	ABHD14A-ACY1 readthrough	-8.361646	0.002036797
100528061	gene-LOC100528061	LOC100528061	gamma-glutamylcyclotransferase pseudogene	-5.204880	0.046195495
100529207	NR_037714	RAD51L3-RFFL	RAD51L3-RFFL readthrough	7.034531	0.001712153
100529211	NR_037719	TMEM256-PLSCR3	TMEM256-PLSCR3 readthrough (NMD candidate)	-10.002254	0.003998158
100532724	NR_037804	NPHP3-ACAD11	NPHP3-ACAD11 readthrough (NMD candidate)	-4.337363	0.015977412
100532731	NM_001204062	COMMD3-BMI1	COMMD3-BMI1 readthrough	5.281043	0.007156609
100533179	NR_037904	UBE2F-SCLY	UBE2F-SCLY readthrough (NMD candidate)	-8.526271	0.000772484
100533496	NM_001204478, NR_037924	TVP23C-CDRT4	TVP23C-CDRT4 readthrough	12.087845	0.000545045
100534595	NR_037946	HNRNPUL2-BSCL2	HNRNPUL2-BSCL2 readthrough (NMD candidate)	6.512618	0.002475992
100885850	NM_001136042, NM_025267	PTGES3L-AARSD1	PTGES3L-AARSD1 readthrough	-3.872460	0.048474468
101926917	NR_136286	ANKRD18CP	ankyrin repeat domain 18C, pseudogene	-8.782682	0.045799632
101927045	NR_110032	LOC101927045	uncharacterized LOC101927045	-4.373708	0.021673081
101927655	NM_001289933	ZASP	ZO-2 associated speckle protein	-11.729110	0.000872758
102725021	NR_157593, NR_157594, NR_157595, NR_157596, NR_157597, NR_157598, NR_157599	LOC102725021	uncharacterized LOC102725021	-5.807880	0.018852638
105274304	NR_130916	LOC105274304	uncharacterized LOC105274304	-4.755217	0.043342751
105377623	NR_136203	LOC105377623	uncharacterized LOC105377623	-7.669975	0.022314819
105378663	NR_135048	LOC105378663	uncharacterized LOC105378663	25.242088	0.002116575
107986827	NR_147989	CZ1P-ASNS	CZ1P-ASNS readthrough	10.506805	0.000833861
109136579	NR_145459	TALAM1	TALAM1 transcript, MALAT1 antisense RNA	4.211655	0.041421069

Table S3 All differentially expressed genes (DEGs) between CotG-p40-treated and wild-type spore-treated HT-29 cells

Gene_ID	Transcript_ID	Gene_symbol	Description	Fold change	*p*-value
220	NM_000693,NM_001293815	ALDH1A3	aldehyde dehydrogenase 1 family member A3	7.576467	0.001083316
868	NM_001321786,NM_001321788,NM_001321789,NM_001321790,NM_001321791,NM_001321793,NM_001321794,NM_001321795,NM_001321796,NM_001321797,NM_001321798,NM_001321799,NM_001321806,NM_001321807,NM_001321808,NM_001321811,NM_001321813,NM_001321816,NM_001321820,NM_001321822,NM_170662,NR_135806,NR_135807,NR_135808,NR_135809,NR_135810,NR_135811,NR_135812	CBLB	Cbl proto-oncogene B	-4.041810	0.02058315
1018	NM_001258	CDK3	cyclin dependent kinase 3	66.017266	2.29661E-05
1026	NM_000389,NM_001220777,NM_001220778,NM_001291549,NM_078467	CDKN1A	cyclin dependent kinase inhibitor 1A	3.820171	0.024966642
1543	NM_000499,NM_001319216,NM_001319217	CYP1A1	cytochrome P450 family 1 subfamily A member 1	247.989966	1.53707E-12
1545	NM_000104	CYP1B1	cytochrome P450 family 1 subfamily B member 1	8.190868	0.000731429
2199	NM_001004019,NM_001165035,NM_001998	FBLN2	fibulin 2	-12.286956	0.005932006
2302	NM_001454	FOXJ1	forkhead box J1	-4.226041	0.027882618
2650	NM_001097633,NM_001097634,NM_001097635,NM_001097636,NM_001490	GCNT1	glucosaminyl (N-acetyl) transferase 1	-3.283405	0.049919922
2702	NM_005266,NM_181703	GJA5	gap junction protein alpha 5	5.184591	0.007348793
2736	NM_001371271,NM_005270	GLI2	GLI family zinc finger 2	4.374530	0.016625784
2886	NM_001030002,NM_001242442,NM_001242443,NM_001330207,NM_005310	GRB7	growth factor receptor bound protein 7	-5.113015	0.007404287

3008	NM_005321	H1-4	H1.4 linker histone, cluster member	4.846372	0.024549514
3009	NM_005322	H1-5	H1.5 linker histone, cluster member	7.303027	0.01012814
3038	NM_001199280,NM_005329,NM_138612	HAS3	hyaluronan synthase 3	5.284968	0.0084257
3137	NR_024240	HLA-J	major histocompatibility complex, class I, J (pseudogene)	5.464061	0.010882272
3352	NM_000864	HTR1D	5-hydroxytryptamine receptor 1D	4.516155	0.012799024
3868	NM_005557	KRT16	keratin 16	4.800764	0.044697393
4242	NM_001166343,NM_002405,NR_029413	MFNG	MFNG O-fucosylpeptide 3-beta-N-acetylglucosaminyltransferase	3.487511	0.043179048
4345	NM_001004196,NM_001318826,NM_001318828,NM_001318830,NM_001365851,NM_001365852,NM_001365853,NM_001365854,NM_001365855,NM_005944,NR_158642	CD200	CD200 molecule	-6.125248	0.023136836
4602	NM_001130172,NM_001130173,NM_001161656,NM_001161657,NM_001161658,NM_001161659,NM_001161660,NM_005375,NR_134958,NR_134959,NR_134960,NR_134961,NR_134962,NR_134963,NR_134964,NR_134965	MYB	MYB proto-oncogene, transcription factor	-3.323644	0.045212548
5013	NM_001199770,NM_014562,NR_130153	OTX1	orthodenticle homeobox 1	-3.837927	0.028115878
5166	NM_002612	PDK4	pyruvate dehydrogenase kinase 4	-5.374822	0.006666929
5582	NM_001316329,NM_002739	PRKCG	protein kinase C gamma	5.076959	0.008975622
5801	NM_001207015,NM_001207016,NM_002849,NM_130846,NR_073474	PTPRR	protein tyrosine phosphatase receptor type R	-4.355911	0.019748177
6029	NR_002715	RN7SL1	RNA component of signal recognition particle 7SL1	4.037074	0.020357397
6364	NM_001130046,NM_004591	CCL20	C-C motif chemokine ligand 20	10.347762	0.000541917
6398	NM_003004	SECTM1	secreted and transmembrane 1	3.469949	0.043254342

6699	NM_003125	SPRR1B	small proline rich protein 1B	5.216325	0.008516244
6707	NM_001097589,NM_005416	SPRR3	small proline rich protein 3	8.395774	0.000923622
6781	NM_003155	STC1	stanniocalcin 1	3.775378	0.047697157
7133	NM_001066	TNFRSF1B	TNF receptor superfamily member 1B	6.953970	0.003119437
7294	NM_003328	TXK	TXK tyrosine kinase	-4.122117	0.025767189
7433	NM_001251882,NM_001251883,NM_001251884,NM_001251885,NM_004624	VIPR1	vasoactive intestinal peptide receptor 1	4.056599	0.019411494
7832	NM_006763	BTG2	BTG anti-proliferation factor 2	-3.554326	0.033979732
8000	NM_005672,NR_033343	PSCA	prostate stem cell antigen	-4.274994	0.018656114
8061	NM_001300844,NM_001300855,NM_001300856,NM_001300857,NM_005438,NR_125339	FOSL1	FOS like 1, AP-1 transcription factor subunit	3.294934	0.044512495
8120	NM_001278511,NM_001278512,NM_001348440,NM_001348441,NM_004644	AP3B2	adaptor related protein complex 3 subunit beta 2	4.448551	0.025839294
8193	NM_001135155,NM_001135156,NM_001289978,NM_001363579,NM_004647	DPF1	double PHD fingers 1	3.698437	0.039440343
8431	NM_021969	NR0B2	nuclear receptor subfamily 0 group B member 2	-4.610779	0.020180045
8654	NM_001083,NM_033430,NM_033437	PDE5A	phosphodiesterase 5A	-3.341985	0.048693612
8743	NM_001190942,NM_001190943,NM_003810,NR_033994	TNFSF10	TNF superfamily member 10	-3.540905	0.036669268
8862	NM_017413	APLN	apelin	13.098585	0.003029173
9832	NM_001270934,NM_001270941,NM_001282282,NM_014790	JAKMIP2	janus kinase and microtubule interacting protein 2	-5.602404	0.034088492
9940	NM_001321153,NM_007335,NM_007337	DLEC1	DLEC1 cilia and flagella associated protein	4.091658	0.023852001

10023	NM_005479	FRAT1	FRAT regulator of WNT signaling pathway 1	-4.114044	0.025838444
10170	NM_001142270,NM_001142271,NM_001289763,NM_199204	DHRS9	dehydrogenase/reductase 9	4.449595	0.014140549
10194	NM_001308210,NM_005786	TSHZ1	teashirt zinc finger homeobox 1	-3.279087	0.046478232
10451	NM_001079874,NM_006113	VAV3	vav guanine nucleotide exchange factor 3	-6.648092	0.018080005
10957	NM_006813	PNRC1	proline rich nuclear receptor coactivator 1	-3.529988	0.037174584
23285	NM_015237	KIAA1107	KIAA1107	-9.896781	0.000742765
23630	NM_012282	KCNE5	potassium voltage-gated channel subfamily E regulatory subunit 5	4.143175	0.043342751
25818	NM_001077491,NM_001077492,NM_012427	KLK5	kallikrein related peptidase 5	6.442320	0.022910824
26959	NM_001244262,NM_012257	HBP1	HMG-box transcription factor 1	-5.190747	0.006907313
27113	NM_001127240,NM_001127241,NM_001127242,NM_014417	BBC3	BCL2 binding component 3	-3.246948	0.049912557
29114	NM_001008272,NM_001008273,NM_013259	TAGLN3	transgelin 3	7.705322	0.001907219
29970	NM_001197107,NM_001197108,NM_001197109,NM_014575	SCHIP1	schwannomin interacting protein 1	-4.196446	0.035521524
51090	NM_015993	PLLP	plasmolipin	4.584056	0.028191532
51676	NM_001202429,NM_016150	ASB2	ankyrin repeat and SOCS box containing 2	6.490694	0.007626368
51702	NM_016233	PADI3	peptidyl arginine deiminase 3	4.874774	0.0105929
54762	NM_001172105,NM_017577	GRAMD1C	GRAM domain containing 1C	-3.809162	0.031397471
54800	NM_001349413,NM_001349414,NM_001349415,NM_001349416,NM_001349417,NM_001349418,NM_001349419,NM_001349420,NM_001349421,NM_001349422,NM_001349423,NM_001349424,NM_00134942	KLHL24	kelch like family member 24	-7.146724	0.001654105

	5,NM_001349426,NM_001349428,NM_001349429,NM_017644,NR_146169,NR_146170				
56204	NM_001286495,NM_019600,NR_104457	FAM214A	family with sequence similarity 214 member A	-4.485459	0.013510008
56670	NM_033050	SUCNR1	succinate receptor 1	-5.227107	0.014570392
56901	NM_020142	NDUFA4L2	NDUFA4 mitochondrial complex associated like 2	20.834670	2.05644E-05
57713	NM_001018039,NM_001029880	SFMBT2	Scm like with four mbt domains 2	4.651806	0.043342751
63982	NM_001313726,NM_001313727,NM_031418	ANO3	anoctamin 3	-3.802730	0.038120537
65986	NM_001105539,NM_001277145,NM_023929	ZBTB10	zinc finger and BTB domain containing 10	-3.605447	0.040906061
79413	NM_024508	ZBED2	zinc finger BED-type containing 2	5.558879	0.010239589
79782	NM_001277127,NM_001277128,NM_024727	LRRC31	leucine rich repeat containing 31	-4.540970	0.029677854
83992	NM_001363349,NM_001363350,NM_001363351,NM_033427	CTTNBP2	cortactin binding protein 2	-4.067982	0.026515575
84159	NM_001244638,NM_032199	ARID5B	AT-rich interaction domain 5B	-4.758960	0.009965108
84189	NM_032229	SLITRK6	SLIT and NTRK like family member 6	-4.614782	0.011484303
85409	NM_001271082,NM_033120	NKD2	NKD inhibitor of WNT signaling pathway 2	6.280439	0.002841804
85495	NR_002312	RPPH1	ribonuclease P RNA component H1	8.423275	0.002346676
89866	NM_001356499,NM_001356500,NM_033127	SEC16B	SEC16 homolog B, endoplasmic reticulum export factor	-4.003197	0.040745019
89884	NM_033343	LHX4	LIM homeobox 4	5.439926	0.007155995
90342	NM_001293083	FER1L5	fer-1 like family member 5	-11.754155	0.016225581
94241	NM_001135733,NM_033285	TP53INP1	tumor protein p53 inducible nuclear	-4.300018	0.020048079

			protein 1		
114814	NR_002328,NR_104033,NR_104034	GNRHR2	gonadotropin releasing hormone receptor 2 (pseudogene)	-4.785082	0.041376294
130574	NM_001195685,NM_194317	LYPD6	LY6/PLAUR domain containing 6	-4.368866	0.028704807
131583	NM_153690	FAM43A	family with sequence similarity 43 member A	6.284641	0.008252859
143686	NM_001271594,NM_144665	SESN3	sestrin 3	-3.367090	0.045699921
145438	NR_037676	FRMD6-AS1	FRMD6 antisense RNA 1	-6.255034	0.036013059
147011	NM_001304949,NM_001304951,NM_001304952,NM_001304953,NM_001304954,NM_001366301,NM_001366302,NM_001366303,NM_152465	PROCA1	protein interacting with cyclin A1	-10.830843	0.002106798
148398	NM_152486	SAMD11	sterile alpha motif domain containing 11	14.769443	0.020717199
148418	NM_001010971,NM_001134663,NM_001134664	SAMD13	sterile alpha motif domain containing 13	-5.749870	0.015534855
148898	NR_033690,NR_033691	ZNF436-AS1	ZNF436 antisense RNA 1	-3.662593	0.040407927
151050	NM_001307976,NM_152519	KANSL1L	KAT8 regulatory NSL complex subunit 1 like	-3.848631	0.025399653
153222	NM_001168393,NM_001168394,NM_153607	CREBRF	CREB3 regulatory factor	-3.835935	0.026201199
154091	NM_145176	SLC2A12	solute carrier family 2 member 12	-4.271835	0.019203166
164312	NM_152611	LRRN4	leucine rich repeat neuronal 4	4.203675	0.019257365
165679	NM_001040100,NM_001320679	SPTSSB	serine palmitoyltransferase small subunit B	-3.485844	0.040344418
219855	NM_001145290,NM_198277	SLC37A2	solute carrier family 37 member 2	3.591660	0.033508238
221091	NM_203422	LRRN4CL	LRRN4 C-terminal like	4.918664	0.049219036
221472	NM_173558	FGD2	FYVE, RhoGEF and PH domain containing 2	4.826943	0.017968629

255488	NM_182757	RNF144B	ring finger protein 144B	-3.944333	0.025096135
267004	NM_170753	PGBD3	piggyBac transposable element derived 3	23.310746	0.00365261
283209	NM_173582	PGM2L1	phosphoglucomutase 2 like 1	-4.516582	0.01316175
283460	NR_024345	HNF1A-AS1	HNF1A antisense RNA 1	-3.454468	0.043809949
283876	NR_033904	LINC00921	long intergenic non-protein coding RNA 921	-5.451687	0.022798603
283897	NM_175900	C16orf54	chromosome 16 open reading frame 54	-11.106392	0.016225581
286204	NM_173689,NR_104603	CRB2	crumbs cell polarity complex component 2	-6.352762	0.005445327
338817	NR_033890	LINC01252	long intergenic non-protein coding RNA 1252	-6.227604	0.011748854
349136	NM_001284260,NM_001284261,NM_001284262,NM_198285	WDR86	WD repeat domain 86	5.733721	0.026707832
374860	NM_001367607,NR_160272	ANKRD30B	ankyrin repeat domain 30B	-7.188034	0.046195495
375791	NM_199001	CYSRT1	cysteine rich tail 1	3.343294	0.046160706
378706	NR_027260	RN7SL2	RNA component of signal recognition particle 7SL2	3.809480	0.025719172
378707	NR_145670	RN7SL3	RNA component of signal recognition particle 7SL3	4.690404	0.026168335
388403	NM_001005404	YPEL2	yippee like 2	-3.667034	0.031204931
389136	NM_001320493,NM_001320494,NM_016206	VGLL3	vestigial like family member 3	-3.434036	0.045568639
389384	NM_001010903	BNIP5	BCL2 interacting protein 5	-6.861282	0.008876868
401251	NM_001039651	SAPCD1	suppressor APC domain containing 1	-4.587088	0.038840397
401303	NR_023382	ZNF815P	zinc finger protein 815, pseudogene	-4.702259	0.023427372
404734	NM_020690	ANKHD1-	ANKHD1-EIF4EBP3 readthrough	3.299804	0.044311623

		EIF4EBP3			
440519	NM_001355404,NM_001355405,NM_001355406	ZNF724	zinc finger protein 724	-5.428122	0.038983062
441386	gene-RPS26P3	RPS26P3	ribosomal protein S26 pseudogene 3	12.329070	0.034780972
445372	NM_001003819	TRIM6-TRIM34	TRIM6-TRIM34 readthrough	5.823760	0.041756881
552891	NM_004125	DNAJC25-GNG10	DNAJC25-GNG10 readthrough	-10.247387	0.012140675
642517	NM_001190810	AGAP9	ArfGAP with GTPase domain, ankyrin repeat and PH domain 9	-6.180367	0.005749243
642956	gene-FABP5P9	FABP5P9	fatty acid binding protein 5 pseudogene 9	5.430662	0.03850259
643596	NM_001190228	RNF224	ring finger protein 224	6.196443	0.015540812
728741	NM_001282524	NPIPB6	nuclear pore complex interacting protein family member B6	5.430662	0.03850259
729291	NR_148936	LOC729291	uncharacterized LOC729291	-4.553190	0.023176648
729438	NM_001145064	CASTOR2	cytosolic arginine sensor for mTORC1 subunit 2	-3.675063	0.032246816
100113386	NR_027287	UCKL1-AS1	UCKL1 antisense RNA 1	4.900765	0.016666963
100128640	NR_028389	ACVR2B-AS1	ACVR2B antisense RNA 1	-11.607100	0.007826126
100131956	gene-RPS3P5	RPS3P5	ribosomal protein S3 pseudogene 5	8.537853	0.041756881
100137049	NM_001114633	PLA2G4B	phospholipase A2 group IVB	18.689189	3.39268E-05
100271594	gene-RPS3AP38	RPS3AP38	ribosomal protein S3a pseudogene 38	-6.485458	0.030962986
100526794	NM_001199103,NM_001199104	NT5C1B-RDH14	NT5C1B-RDH14 readthrough	3.388987	0.047598925
100526836	NR_037616	BLOC1S5-TXNDC5	BLOC1S5-TXNDC5 readthrough (NMD candidate)	16.650170	0.000165853

100529264	NR_037791	RAB4B-EGLN2	RAB4B-EGLN2 readthrough (NMD candidate)	8.462432	0.001188224
100532736	NM_001204088,NM_001204089	MICOS10-NBL1	MICOS10-NBL1 readthrough	40.323141	1.22351E-06
100532737	NR_037853	ATP6V1G2-DDX39B	ATP6V1G2-DDX39B readthrough (NMD candidate)	-5.247656	0.006859141
100533179	NR_037904	UBE2F-SCLY	UBE2F-SCLY readthrough (NMD candidate)	19.593866	0.000146292
100534592	NM_001204871	URGCP-MRPS24	URGCP-MRPS24 readthrough	-5.993244	0.014138806
100534595	NR_037946	HNRNPUL2-BSCL2	HNRNPUL2-BSCL2 readthrough (NMD candidate)	7.944027	0.00094139
100873955	NR_046827	LNX1-AS2	LNX1 antisense RNA 2	-8.547747	0.027120138
101927830	NR_109985	LOC101927830	uncharacterized LOC101927830	-5.794186	0.049219036
101928837	NR_120561,NR_120562	LOC101928837	uncharacterized LOC101928837	-4.352728	0.043342751
101928991	NM_001355573	CCDC92B	coiled-coil domain containing 92B	-4.938627	0.028229227
101929125	NR_109859	LINC01730	long intergenic non-protein coding RNA 1730	5.767881	0.03224147
102725021	NR_157593,NR_157594,NR_157595,NR_157596,NR_157597,NR_157598,NR_157599	LOC102725021	uncharacterized LOC102725021	-5.524525	0.018852638
105378663	NR_135048	LOC105378663	uncharacterized LOC105378663	6.277092	0.020057392
105378828	NR_135837,NR_135838	CLCA4-AS1	CLCA4 antisense RNA 1	-3.665426	0.043436716
106633809	NR_132760	SNORD144	small nucleolar RNA, C/D box 144	-8.515337	0.045799632

Table S4 All differentially expressed genes (DEGs) between wild-type spore treated HT-29 cells and control

Gene_ID	Transcript_ID	Gene_Symbol	Description	Fold change	*p*-value
800	NM_004342, NM_033138, NM_033139, NM_033140, NM_033157	CALD1	caldesmon 1	4.025590	0.021927298
2297	NM_004472	FOXD1	forkhead box D1	-6.833515	0.02310992
2650	NM_001097633, NM_001097634, NM_001097635, NM_001097636, NM_001490	GCNT1	glucosaminyl (N-acetyl) transferase 1	4.040295	0.022130738
2833	NM_001142797, NM_001504	CXCR3	C-X-C motif chemokine receptor 3	-5.502045	0.038983062
3013	NM_021065	H2AC7	H2A clustered histone 7	-5.521472	0.028984011
3112	NM_002120	HLA-DOB	major histocompatibility complex, class II, DO beta	4.715801	0.038983062
4435	NM_001144885, NM_001144886, NM_001144887, NM_004143	CITED1	Cbp/p300 interacting transactivator with Glu/Asp rich carboxy-terminal domain 1	-8.297304	0.016551802
5320	NM_000300, NM_001161727, NM_001161728, NM_001161729	PLA2G2A	phospholipase A2 group IIA	-4.076963	0.044985511
6262	NM_001035	RYR2	ryanodine receptor 2	4.996985	0.042028125
6696	NM_000582, NM_001040058, NM_001040060, NM_001251829, NM_001251830	SPP1	secreted phosphoprotein 1	4.692928	0.01079878
6818	NM_177552	SULT1A3	sulfotransferase family 1A member 3	4.548766	0.017178121
7125	NM_003279	TNNC2	troponin C2, fast skeletal type	-4.076963	0.044985511
7366	NM_001076	UGT2B15	UDP glucuronosyltransferase family 2 member B15	5.861055	0.02310992
8367	NM_003545	H4C5	H4 clustered histone 5	-4.854778	0.025461672
8557	NM_003673	TCAP	titin-cap	-5.338143	0.032569892
9021	NM_003955	SOCS3	suppressor of cytokine signaling 3	4.583194	0.032799238
9148	NM_004210	NEURL1	neuralized E3 ubiquitin protein ligase 1	-6.769473	0.004547879

10538	NM_006399	BATF	basic leucine zipper ATF-like transcription factor	-5.502045	0.038983062
10603	NM_001359228, NM_001359229, NM_001359230, NM_001359231, NM_020979	SH2B2	SH2B adaptor protein 2	-4.152415	0.040082012
25758	NM_012194	KIAA1549L	KIAA1549 like	9.657069	0.001766028
27031	NM_153240	NPHP3	nephrocystin 3	6.854773	0.00205898
27099	NR_027330	SND1-IT1	SND1 intronic transcript 1	9.655283	0.045799632
29887	NM_001199835, NM_001199837, NM_001199838, NM_001318198, NM_001318199, NM_001362753, NM_001362754, NM_013322	SNX10	sorting nexin 10	4.798461	0.016201814
43849	NM_001370125, NM_001370126, NM_001370127, NM_001370128, NM_019598, NM_145894, NM_145895	KLK12	kallikrein related peptidase 12	-3.575799	0.04469104
51208	NM_001002026, NM_016369	CLDN18	claudin 18	6.285487	0.003100792
55704	NM_001135597, NM_001254943, NM_001365480, NM_018084	CCDC88A	coiled-coil domain containing 88A	3.486537	0.049561987
56901	NM_020142	NDUFA4L2	NDUFA4 mitochondrial complex associated like 2	-15.160166	9.86186E-05
57393	NM_020665	CLTRN	collectrin, amino acid transport regulator	4.996985	0.042028125
57731	NM_020971, NM_025213	SPTBN4	spectrin beta, non-erythrocytic 4	-7.970625	0.033664757
80125	NM_001287181, NM_025055, NM_182791	CCDC33	coiled-coil domain containing 33	4.765371	0.040683222
81796	NM_001146008, NM_001146009, NM_030958	SLCO5A1	solute carrier organic anion transporter family member 5A1	-3.902819	0.048605977
84129	NM_032169, NR_132426, NR_132427, NR_132428	ACAD11	acyl-CoA dehydrogenase family member 11	9.028026	0.000688367
84525	NM_001145459, NM_001145460, NM_032495, NM_139211, NM_139212	HOPX	HOP homeobox	3.815231	0.044445535
114814	NR_002328, NR_104033, NR_104034	GNRHR2	gonadotropin releasing hormone receptor 2 (pseudogene)	4.962732	0.028984011

131583	NM_153690	FAM43A	family with sequence similarity 43 member A	-4.859335	0.021003933
149111	NM_001322302, NM_001322303, NM_001322304, NM_001322305, NM_152495, NR_136287, NR_136288, NR_136289, NR_136290, NR_136291, NR_136292, NR_136293, NR_136294, NR_136295, NR_136296, NR_136297	CNIH3	cornichon family AMPA receptor auxiliary protein 3	6.599769	0.022910824
222901	NR_026673	RPL23P8	ribosomal protein L23 pseudogene 8	-4.354392	0.033908436
253012	NM_001039372, NM_001288804, NM_001288810, NM_001346642, NM_198151	HEPACAM2	HEPACAM family member 2	-4.250592	0.029260307
285888	NM_001103176, NM_001369813, NM_001369814, NM_001369815, NM_001369816, NM_001369818, NM_001369820, NM_001369821, NR_163157, NR_163158, NR_163159, NR_163160, NR_163161, NR_163162, NR_163163, NR_163164, NR_163165, NR_163166, NR_163167	CNPY1	canopy FGF signaling regulator 1	-8.497863	0.027322057
326321	gene-RPS24P2	RPS24P2	ribosomal protein S24 pseudogene 2	-9.552339	0.018334706
340547	NM_001170553, NM_182607	VSIG1	V-set and immunoglobulin domain containing 1	4.500997	0.01431428
387522	NM_199203	TMEM189-UBE2V1	TMEM189-UBE2V1 readthrough	4.783724	0.013097463
406984	NR_029639, rna-MIR200B, rna-MIR200B-2	MIR200B	microRNA 200b	-4.971483	0.041376294
552891	NM_004125	DNAJC25-GNG10	DNAJC25-GNG10 readthrough	12.656475	0.003915642
643932	gene-RPS3AP20	RPS3AP20	ribosomal protein S3a pseudogene 20	-5.502045	0.038983062
728202	gene-RPL36AP7	RPL36AP7	ribosomal protein L36a pseudogene 7	-6.098966	0.009812054
728324	gene-CHCHD2P2	CHCHD2P2	coiled-coil-helix-coiled-coil-helix domain containing 2 pseudogene 2	-6.861641	0.03224147
729779	gene-PSAT1P3	PSAT1P3	phosphoserine aminotransferase 1 pseudogene 3	8.036145	0.022314819
100132686	NR_133566	LOC100132686	uncharacterized LOC100132686	12.042779	0.020717199
100137049	NM_001114633	PLA2G4B	phospholipase A2 group IVB	-24.113237	6.31304E-06

100270862	gene-RPS3P4	RPS3P4	ribosomal protein S3 pseudogene 4	-6.691625	0.004863127
100271594	gene-RPS3AP38	RPS3AP38	ribosomal protein S3a pseudogene 38	6.077072	0.02310992
100271603	gene-RPL13AP24	RPL13AP24	ribosomal protein L13a pseudogene 24	-12.002652	0.001942282
100421350	gene-TCEA1P4	TCEA1P4	transcription elongation factor A1 pseudogene 4	4.962732	0.028984011
100422495	gene-LOC100422495	LOC100422495	protein tyrosine phosphatase non-receptor type 4 pseudogene	6.029159	0.03224147
100422595	gene-PCMTD1P3	PCMTD1P3	protein-L-isoaspartate (D-aspartate) O-methyltransferase domain containing 1 pseudogene 3	-7.443386	0.041756881
100506055	gene-LOC100506055	LOC100506055	matrix remodelling associated 7 pseudogene	-6.561455	0.026707832
100506207	NR_038979, NR_038980	LOC100506207	uncharacterized LOC100506207	-6.143809	0.046195495
100507487	NR_125882	LINC02615	long intergenic non-protein coding RNA 2615	9.716265	0.010510413
100526760	NM_001316331	ABHD14A-ACY1	ABHD14A-ACY1 readthrough	-11.663388	0.000423598
100526836	NR_037616	BLOC1S5-TXNDC5	BLOC1S5-TXNDC5 readthrough (NMD candidate)	-5.535452	0.025461672
100529067	NR_037673	SERF2-C15ORF63	SERF2-C15orf63 readthrough	-4.873323	0.00971624
100529207	NR_037714	RAD51L3-RFFL	RAD51L3-RFFL readthrough	6.056517	0.003462198
100529211	NR_037719	TMEM256-PLSCR3	TMEM256-PLSCR3 readthrough (NMD candidate)	-3.797724	0.047710877
100529264	NR_037791	RAB4B-EGLN2	RAB4B-EGLN2 readthrough (NMD candidate)	-18.959812	1.81656E-05
100532724	NR_037804	NPHP3-ACAD11	NPHP3-ACAD11 readthrough (NMD candidate)	-12.293952	0.000110881
100532731	NM_001204062	COMMD3-BMI1	COMMD3-BMI1 readthrough	5.930134	0.00422247
100532736	NM_001204088, NM_001204089	MICOS10-NBL1	MICOS10-NBL1 readthrough	-13.259114	0.00051512
100532737	NR_037853	ATP6V1G2-DDX39B	ATP6V1G2-DDX39B readthrough (NMD candidate)	6.090238	0.003447877
100533179	NR_037904	UBE2F-SCLY	UBE2F-SCLY readthrough (NMD candidate)	-167.620884	7.68645E-10
100533496	NM_001204478, NR_037924	TVP23C-CDRT4	TVP23C-CDRT4 readthrough	12.396435	0.000509863

100874146	NR_046998	LINC00365	long intergenic non-protein coding RNA 365	5.429020	0.030962986
101243545	NR_102265	LINC02067	long intergenic non-protein coding RNA 2067	4.416536	0.030346688
101927655	NM_001289933	ZASP	ZO-2 associated speckle protein	-46.986258	9.49475E-06
106633808	NR_132759	SNORD143	small nucleolar RNA, C/D box 143	5.237223	0.029884417

Table S5 Gene ontology (GO) enrichment analysis of 163 differentially expressed genes (DEGs) between CotG-p40-treated HT-29 cells and control

GO ID	GO Description	*p*-value	Corrected *p*-value[a]	Cluster frequency[b]	Total frequency[c]	Genes
48731	system development	8.99E-06	9.04E-03	32/107 29.9%	2422/17785 13.6%	*SPRR3 SEMA7A CDKN1A PTPRR BTG2 GFI1 TSHZ1 SECTM1 PTGS2 GLI2 DLL4 GJA5 SPP1 SLITRK6 OTX1 MSX1 KIRREL3 EDN1 FOXF1 LRRN4 ARID5B EREG TGFBR3 ALDH1A3 BCL6 DHRS9 CYP1A1 MMP19 TAGLN3 SPRR1A MDGA1 SPRR1B*
42471	ear morphogenesis	4.79E-05	1.97E-02	5/107 4.6%	66/17785 0.3%	*EDN1 GFI1 TSHZ1 OTX1 GLI2*
34754	cellular hormone metabolic process	4.79E-05	1.97E-02	5/107 4.6%	66/17785 0.3%	*ALDH1A3 DHRS9 HSD3B1 CYP1A1 CYP1B1*
9913	epidermal cell differentiation	5.53E-05	1.97E-02	5/107 4.6%	68/17785 0.3%	*SPRR3 SPRR1A SPRR1B EREG GLI2*
48856	anatomical structure development	5.87E-05	1.97E-02	32/107 29.9%	2656/17785 14.9%	*SPRR3 SEMA7A CDKN1A PTPRR BTG2 GFI1 TSHZ1 SECTM1 PTGS2 GLI2 DLL4 GJA5 SPP1 SLITRK6 OTX1 MSX1 KIRREL3 EDN1 FOXF1 LRRN4 ARID5B EREG TGFBR3 ALDH1A3 BCL6 DHRS9 CYP1A1 MMP19 TAGLN3 SPRR1A MDGA1 SPRR1B*
8285	negative regulation of cell proliferation	9.62E-05	2.26E-02	10/107 9.3%	379/17785 2.1%	*TGFBR3 CDKN1A BTG2 BCL6 CBLB DLEC1 MSX1 PTGS2 EREG PTGES*
90068	positive regulation of cell cycle process	1.28E-04	2.26E-02	4/107 3.7%	43/17785 0.2%	*CDKN1A EDN1 CYP1A1 EREG*
42573	retinoic acid metabolic process	1.35E-04	2.26E-02	3/107 2.8%	17/17785 0.0%	*ALDH1A3 DHRS9 CYP1A1*

9888	tissue development	1.60E-04	2.26E-02	14/107 13.0%	750/17785 4.2%	*SPRR3 EDN1 FOXF1 SECTM1 PTGS2 EREG GLI2 TGFBR3 ALDH1A3 DHRS9 SPP1 MSX1 SPRR1A SPRR1B*
30728	ovulation	1.62E-04	2.26E-02	3/107 2.8%	18/17785 0.1%	*MMP19 PTGS2 EREG*
48598	embryonic morphogenesis	1.95E-04	2.26E-02	9/107 8.4%	336/17785 1.8%	*ALDH1A3 EDN1 FOXF1 GFI1 GJA5 TSHZ1 OTX1 MSX1 GLI2*
60429	epithelium development	1.99E-04	2.26E-02	9/107 8.4%	337/17785 1.8%	*ALDH1A3 SPRR3 EDN1 DHRS9 FOXF1 SPRR1A SPRR1B EREG GLI2*
48562	embryonic organ morphogenesis	2.01E-04	2.26E-02	6/107 5.6%	140/17785 0.7%	*ALDH1A3 EDN1 GFI1 TSHZ1 OTX1 GLI2*
7166	cell surface receptor linked signaling pathway	2.09E-04	2.26E-02	19/107 17.7%	1280/17785 7.1%	*PTGER4 GRB7 GPR17 VIPR1 EDN1 FOXF1 NPY1R ARID5B CBLB TNFRSF1B MTSS1 EREG GLI2 NKD2 TGFBR3 DLL4 FRAT1 HBP1 MSX1*
42904	9-cis-retinoic acid biosynthetic process	2.13E-04	2.26E-02	2/107 1.8%	4/17785 0.0%	*DHRS9 CYP1A1*
42905	9-cis-retinoic acid metabolic process	2.13E-04	2.26E-02	2/107 1.8%	4/17785 0.0%	*DHRS9 CYP1A1*
35238	vitamin A biosynthetic process	2.13E-04	2.26E-02	2/107 1.8%	4/17785 0.0%	*DHRS9 CYP1A1*
7275	multicellular organismal development	2.14E-04	2.26E-02	33/107 30.8%	2971/17785 16.7%	*SPRR3 SEMA7A CDKN1A PTPRR BTG2 GFI1 TSHZ1 SECTM1 PTGS2 GLI2 DLL4 FRAT1 GJA5 SPP1 SLITRK6 OTX1 MSX1 KIRREL3 EDN1 FOXF1 LRRN4 ARID5B EREG TGFBR3 ALDH1A3 BCL6 DHRS9 CYP1A1 MMP19 TAGLN3 SPRR1A MDGA1 SPRR1B*

6692	prostanoid metabolic process	2.60E-04	2.41E-02	3/107 2.8%	21/17785 0.1%	*EDN1 PTGS2 PTGES*
6693	prostaglandin metabolic process	2.60E-04	2.41E-02	3/107 2.8%	21/17785 0.1%	*EDN1 PTGS2 PTGES*
9790	embryonic development	2.64E-04	2.41E-02	12/107 11.2%	601/17785 3.3%	*ALDH1A3 FRAT1 PTPRR EDN1 FOXF1 GFI1 GJA5 TSHZ1 CYP1A1 OTX1 MSX1 GLI2*
48568	embryonic organ development	3.21E-04	2.72E-02	7/107 6.5%	215/17785 1.2%	*ALDH1A3 EDN1 FOXF1 GFI1 TSHZ1 OTX1 GLI2*
10817	regulation of hormone levels	3.25E-04	2.72E-02	6/107 5.6%	153/17785 0.8%	*ALDH1A3 EDN1 DHRS9 HSD3B1 CYP1A1 CYP1B1*
42362	fat-soluble vitamin biosynthetic process	3.54E-04	2.78E-02	2/107 1.8%	5/17785 0.0%	*DHRS9 CYP1A1*
43583	ear development	3.59E-04	2.78E-02	5/107 4.6%	101/17785 0.5%	*EDN1 GFI1 TSHZ1 OTX1 GLI2*
9887	organ morphogenesis	4.34E-04	3.23E-02	12/107 11.2%	635/17785 3.5%	*TGFBR3 ALDH1A3 CDKN1A EDN1 FOXF1 GFI1 TSHZ1 ARID5B OTX1 MSX1 EREG GLI2*
19748	secondary metabolic process	4.96E-04	3.53E-02	4/107 3.7%	61/17785 0.3%	*ALDH1A3 DHRS9 CYP1A1 CYP1B1*
42445	hormone metabolic process	5.32E-04	3.53E-02	5/107 4.6%	110/17785 0.6%	*ALDH1A3 DHRS9 HSD3B1 CYP1A1 CYP1B1*
30855	epithelial cell differentiation	5.34E-04	3.53E-02	6/107 5.6%	168/17785 0.9%	*SPRR3 DHRS9 FOXF1 SPRR1A SPRR1B EREG*
42127	regulation of cell proliferation	5.55E-04	3.53E-02	14/107 13.0%	848/17785 4.7%	*VIPR1 CDKN1A BTG2 EDN1 FOXF1 CBLB DLEC1 PTGS2 EREG GLI2 TGFBR3 BCL6 MSX1 PTGES*
30216	keratinocyte differentiation	5.62E-04	3.53E-02	4/107 3.7%	63/17785 0.3%	*SPRR3 SPRR1A SPRR1B EREG*

60485	mesenchyme development	5.96E-04	3.60E-02	4/107 3.7%	64/17785 0.3%	*TGFBR3 EDN1 FOXF1 MSX1*
9110	vitamin biosynthetic process	6.22E-04	3.60E-02	3/107 2.8%	28/17785 0.1%	*DHRS9 CYP1A1 CBLB*
1523	retinoid metabolic process	6.90E-04	3.60E-02	3/107 2.8%	29/17785 0.1%	*ALDH1A3 DHRS9 CYP1A1*
6776	vitamin A metabolic process	6.90E-04	3.60E-02	3/107 2.8%	29/17785 0.1%	*ALDH1A3 DHRS9 CYP1A1*
48661	positive regulation of smooth muscle cell proliferation	6.90E-04	3.60E-02	3/107 2.8%	29/17785 0.1%	*EDN1 PTGS2 EREG*
16101	diterpenoid metabolic process	6.90E-04	3.60E-02	3/107 2.8%	29/17785 0.1%	*ALDH1A3 DHRS9 CYP1A1*
10033	response to organic substance	7.06E-04	3.60E-02	14/107 13.0%	869/17785 4.8%	*CDKN1A BTG2 FOXF1 NPY1R PTGS2 EREG TGFBR3 SELL CYP1A1 SPP1 CYP1B1 MMP19 CHAC1 MSX1*
7423	sensory organ development	7.17E-04	3.60E-02	7/107 6.5%	246/17785 1.3%	*ALDH1A3 EDN1 GFI1 TSHZ1 CYP1A1 OTX1 GLI2*
9404	toxin metabolic process	7.38E-04	3.62E-02	2/107 1.8%	7/17785 0.0%	*CYP1A1 CYP1B1*
30154	cell differentiation	8.55E-04	4.09E-02	21/107 19.6%	1668/17785 9.3%	*SPRR3 SEMA7A PTPRR BTG2 EDN1 FOXF1 GFI1 EREG GLI2 TGFBR3 DLL4 BCL6 DHRS9 CYP1A1 SPP1 SLITRK6 MMP19 MSX1 SPRR1A MDGA1 SPRR1B*
8544	epidermis development	9.10E-04	4.26E-02	6/107 5.6%	186/17785 1.0%	*SPRR3 PTGS2 SPRR1A SPRR1B EREG GLI2*
9653	anatomical structure morphogenesis	9.37E-04	4.28E-02	17/107 15.8%	1218/17785 6.8%	*CDKN1A EDN1 FOXF1 GFI1 TSHZ1 ARID5B EREG GLI2 TGFBR3 DLL4 ALDH1A3 BCL6 GJA5 SLITRK6 MMP19 OTX1 MSX1*

32502	developmental process	1.02E-03	4.56E-02	33/107 30.8%	3234/17785 18.1%	*SPRR3 SEMA7A CDKN1A PTPRR BTG2 GFI1 TSHZ1 SECTM1 PTGS2 GLI2 DLL4 FRAT1 GJA5 SPP1 SLITRK6 OTX1 MSX1 KIRREL3 EDN1 FOXF1 LRRN4 ARID5B EREG TGFBR3 ALDH1A3 BCL6 DHRS9 CYP1A1 MMP19 TAGLN3 SPRR1A MDGA1 SPRR1B*
42221	response to chemical stimulus	1.10E-03	4.82E-02	19/107 17.7%	1465/17785 8.2%	*RTP4 CDKN1A BTG2 EDN1 FOXF1 CCL20 NPY1R PTGS2 EREG TGFBR3 ALDH1A3 SELL CYP1A1 SPP1 CYP1B1 MMP19 CHAC1 MSX1 NEIL1*

[a] Corrected *p*-value: After correction, the *p* value in the hypergeometric test

[b] Cluster frequency: the numerator represents the number of each GO term genes and the denominator represents the total number of genes with GO annotation.

[c] Total frequency: the numerator represents the number of reference genes annotated in the listed GO term and the denominator represents the number of reference genes with GO annotation.

Table S6 Gene ontology (GO) enrichment analysis of 147 differentially expressed genes (DEGs) between CotG-p40- and wild-type spore-treated HT-29 cells

GO ID	GO Description	*p*-value	Corrected *p*-value[a]	Cluster frequency[b]	Total frequency[c]	Genes
48731	system development	7.38E-06	7.71E-03	28/72 38.8%	2422/14303 16.9%	*SPRR3 CDKN1A PTPRR BTG2 TSHZ1 KLK5 STC1 SECTM1 GLI2 GJA5 DPF1 SLITRK6 OTX1 VAV3 PLLP LRRN4 ARID5B FOXJ1 NR0B2 APLN ALDH1A3 DHRS9 KRT16 CYP1A1 PDE5A TAGLN3 LHX4 SPRR1B*
48856	anatomical structure development	4.33E-05	1.18E-02	28/72 38.8%	2656/14303 18.5%	*SPRR3 CDKN1A PTPRR BTG2 TSHZ1 KLK5 STC1 SECTM1 GLI2 GJA5 DPF1 SLITRK6 OTX1 VAV3 PLLP LRRN4 ARID5B FOXJ1 NR0B2 APLN ALDH1A3 DHRS9 KRT16 CYP1A1 PDE5A TAGLN3 LHX4 SPRR1B*
7275	multicellular organismal development	4.53E-05	1.18E-02	30/72 41.6%	2971/14303 20.7%	*SPRR3 CDKN1A PTPRR BTG2 TSHZ1 KLK5 STC1 SECTM1 GLI2 FRAT1 GJA5 DPF1 SLITRK6 OTX1 VAV3 PLLP LRRN4 ARID5B FOXJ1 NR0B2 APLN ALDH1A3 DHRS9 KRT16 MFNG CYP1A1 PDE5A TAGLN3 LHX4 SPRR1B*
43065	positive regulation of apoptosis	6.92E-05	1.18E-02	10/72 13.8%	442/14303 3.0%	*FOSL1 VAV3 ALDH1A3 CDKN1A DPF1 TP53INP1 TNFSF10 PDE5A BBC3 FGD2*
43068	positive regulation of programmed cell death	7.33E-05	1.18E-02	10/72 13.8%	445/14303 3.1%	*FOSL1 VAV3 ALDH1A3 CDKN1A DPF1 TP53INP1 TNFSF10 PDE5A BBC3 FGD2*
10942	positive regulation of cell death	7.89E-05	1.18E-02	10/72 13.8%	449/14303 3.1%	*FOSL1 VAV3 ALDH1A3 CDKN1A DPF1 TP53INP1 TNFSF10 PDE5A BBC3 FGD2*
42573	retinoic acid metabolic process	7.91E-05	1.18E-02	3/72 4.1%	17/14303 0.1%	*ALDH1A3 DHRS9 CYP1A1*
35238	vitamin A biosynthetic process	1.49E-04	1.56E-02	2/72 2.7%	4/14303 0.0%	*DHRS9 CYP1A1*
42904	9-cis-retinoic acid biosynthetic	1.49E-04	1.56E-02	2/72 2.7%	4/14303 0.0%	*DHRS9 CYP1A1*

	process					
42905	9-cis-retinoic acid metabolic process	1.49E-04	1.56E-02	2/72 2.7%	4/14303 0.0%	*DHRS9 CYP1A1*
32502	developmental process	2.31E-04	1.99E-02	30/72 41.6%	3234/14303 22.6%	*SPRR3 CDKN1A PTPRR BTG2 TSHZ1 KLK5 STC1 SECTM1 GLI2 FRAT1 GJA5 DPF1 SLITRK6 OTX1 VAV3 PLLP LRRN4 ARID5B FOXJ1 NR0B2 APLN ALDH1A3 DHRS9 KRT16 MFNG CYP1A1 PDE5A TAGLN3 LHX4 SPRR1B*
42362	fat-soluble vitamin biosynthetic process	2.47E-04	1.99E-02	2/72 2.7%	5/14303 0.0%	*DHRS9 CYP1A1*
19748	secondary metabolic process	2.48E-04	1.99E-02	4/72 5.5%	61/14303 0.4%	*ALDH1A3 DHRS9 CYP1A1 CYP1B1*
34754	cellular hormone metabolic process	3.36E-04	2.30E-02	4/72 5.5%	66/14303 0.4%	*ALDH1A3 DHRS9 CYP1A1 CYP1B1*
9110	vitamin biosynthetic process	3.66E-04	2.30E-02	3/72 4.1%	28/14303 0.1%	*DHRS9 CYP1A1 CBLB*
6776	vitamin A metabolic process	4.07E-04	2.30E-02	3/72 4.1%	29/14303 0.2%	*ALDH1A3 DHRS9 CYP1A1*
1523	retinoid metabolic process	4.07E-04	2.30E-02	3/72 4.1%	29/14303 0.2%	*ALDH1A3 DHRS9 CYP1A1*
16101	diterpenoid metabolic process	4.07E-04	2.30E-02	3/72 4.1%	29/14303 0.2%	*ALDH1A3 DHRS9 CYP1A1*
7389	pattern specification process	4.18E-04	2.30E-02	7/72 9.7%	271/14303 1.8%	*FRAT1 BTG2 MFNG TSHZ1 FOXJ1 OTX1 GLI2*
2643	regulation of tolerance induction	5.16E-04	2.45E-02	2/72 2.7%	7/14303 0.0%	*FOXJ1 CBLB*
2645	positive regulation of tolerance induction	5.16E-04	2.45E-02	2/72 2.7%	7/14303 0.0%	*FOXJ1 CBLB*
9404	toxin metabolic process	5.16E-04	2.45E-02	2/72 2.7%	7/14303 0.0%	*CYP1A1 CYP1B1*
6766	vitamin metabolic process	7.33E-04	3.33E-02	4/72 5.5%	81/14303 0.5%	*ALDH1A3 DHRS9 CYP1A1 CBLB*
6721	terpenoid metabolic process	8.41E-04	3.46E-02	3/72 4.1%	37/14303 0.2%	*ALDH1A3 DHRS9 CYP1A1*
46685	response to arsenic	8.79E-04	3.46E-02	2/72 2.7%	9/14303 0.0%	*CDKN1A CYP1A1*

6775	fat-soluble vitamin metabolic process	9.82E-04	3.46E-02	3/72 4.1%	39/14303 0.2%	*ALDH1A3 DHRS9 CYP1A1*
32501	multicellular organismal process	9.95E-04	3.46E-02	35/72 48.6%	4375/14303 30.5%	*SPRR3 VIPR1 CDKN1A PTPRR BTG2 TSHZ1 KLK5 STC1 SECTM1 GLI2 FRAT1 GJA5 DPF1 SLITRK6 CYP1B1 OTX1 PRKCG VAV3 PLLP HTR1D LRRN4 ARID5B FOXJ1 NR0B2 APLN FOSL1 ALDH1A3 DHRS9 KRT16 MFNG CYP1A1 PDE5A TAGLN3 LHX4 SPRR1B*
6917	induction of apoptosis	1.02E-03	3.46E-02	7/72 9.7%	315/14303 2.2%	*VAV3 CDKN1A DPF1 TP53INP1 TNFSF10 BBC3 FGD2*
12502	induction of programmed cell death	1.04E-03	3.46E-02	7/72 9.7%	316/14303 2.2%	*VAV3 CDKN1A DPF1 TP53INP1 TNFSF10 BBC3 FGD2*
42981	regulation of apoptosis	1.04E-03	3.46E-02	12/72 16.6%	852/14303 5.9%	*FOSL1 VAV3 ALDH1A3 CDKN1A BTG2 DPF1 TP53INP1 TNFSF10 PDE5A LHX4 BBC3 FGD2*
48513	organ development	1.08E-03	3.46E-02	19/72 26.3%	1792/14303 12.5%	*VAV3 SPRR3 CDKN1A TSHZ1 KLK5 ARID5B SECTM1 NR0B2 GLI2 APLN ALDH1A3 DHRS9 KRT16 GJA5 CYP1A1 TAGLN3 OTX1 LHX4 SPRR1B*
7567	parturition	1.10E-03	3.46E-02	2/72 2.7%	10/14303 0.0%	*PLA2G4B CYP1A1*
55093	response to hyperoxia	1.10E-03	3.46E-02	2/72 2.7%	10/14303 0.0%	*CDKN1A CYP1A1*
43067	regulation of programmed cell death	1.12E-03	3.46E-02	12/72 16.6%	860/14303 6.0%	*FOSL1 VAV3 ALDH1A3 CDKN1A BTG2 DPF1 TP53INP1 TNFSF10 PDE5A LHX4 BBC3 FGD2*
10941	regulation of cell death	1.21E-03	3.60E-02	12/72 16.6%	867/14303 6.0%	*FOSL1 VAV3 ALDH1A3 CDKN1A BTG2 DPF1 TP53INP1 TNFSF10 PDE5A LHX4 BBC3 FGD2*
48731	system development	7.38E-06	7.71E-03	28/72 38.8%	2422/14303 16.9%	*SPRR3 CDKN1A PTPRR BTG2 TSHZ1 KLK5 STC1 SECTM1 GLI2 GJA5 DPF1 SLITRK6 OTX1 VAV3 PLLP LRRN4*

						ARID5B FOXJ1 NR0B2 APLN ALDH1A3 DHRS9 KRT16 CYP1A1 PDE5A TAGLN3 LHX4 SPRR1B
48856	anatomical structure development	4.33E-05	1.18E-02	28/72 38.8%	2656/14303 18.5%	*SPRR3 CDKN1A PTPRR BTG2 TSHZ1 KLK5 STC1 SECTM1 GLI2 GJA5 DPF1 SLITRK6 OTX1 VAV3 PLLP LRRN4 ARID5B FOXJ1 NR0B2 APLN ALDH1A3 DHRS9 KRT16 CYP1A1 PDE5A TAGLN3 LHX4 SPRR1B*
7275	multicellular organismal development	4.53E-05	1.18E-02	30/72 41.6%	2971/14303 20.7%	*SPRR3 CDKN1A PTPRR BTG2 TSHZ1 KLK5 STC1 SECTM1 GLI2 FRAT1 GJA5 DPF1 SLITRK6 OTX1 VAV3 PLLP LRRN4 ARID5B FOXJ1 NR0B2 APLN ALDH1A3 DHRS9 KRT16 MFNG CYP1A1 PDE5A TAGLN3 LHX4 SPRR1B*
43065	positive regulation of apoptosis	6.92E-05	1.18E-02	10/72 13.8%	442/14303 3.0%	*FOSL1 VAV3 ALDH1A3 CDKN1A DPF1 TP53INP1 TNFSF10 PDE5A BBC3 FGD2*
43068	positive regulation of programmed cell death	7.33E-05	1.18E-02	10/72 13.8%	445/14303 3.1%	*FOSL1 VAV3 ALDH1A3 CDKN1A DPF1 TP53INP1 TNFSF10 PDE5A BBC3 FGD2*
10942	positive regulation of cell death	7.89E-05	1.18E-02	10/72 13.8%	449/14303 3.1%	*FOSL1 VAV3 ALDH1A3 CDKN1A DPF1 TP53INP1 TNFSF10 PDE5A BBC3 FGD2*
42573	retinoic acid metabolic process	7.91E-05	1.18E-02	3/72 4.1%	17/14303 0.1%	*ALDH1A3 DHRS9 CYP1A1*
35238	vitamin A biosynthetic process	1.49E-04	1.56E-02	2/72 2.7%	4/14303 0.0%	*DHRS9 CYP1A1*
42904	9-cis-retinoic acid biosynthetic process	1.49E-04	1.56E-02	2/72 2.7%	4/14303 0.0%	*DHRS9 CYP1A1*
42905	9-cis-retinoic acid metabolic process	1.49E-04	1.56E-02	2/72 2.7%	4/14303 0.0%	*DHRS9 CYP1A1*
32502	developmental process	2.31E-04	1.99E-02	30/72 41.6%	3234/14303 22.6%	*SPRR3 CDKN1A PTPRR BTG2 TSHZ1 KLK5 STC1 SECTM1 GLI2 FRAT1 GJA5 DPF1 SLITRK6 OTX1 VAV3 PLLP LRRN4 ARID5B FOXJ1 NR0B2 APLN ALDH1A3 DHRS9 KRT16 MFNG CYP1A1 PDE5A*

						TAGLN3 LHX4 SPRR1B
42362	fat-soluble vitamin biosynthetic process	2.47E-04	1.99E-02	2/72 2.7%	5/14303 0.0%	*DHRS9 CYP1A1*
19748	secondary metabolic process	2.48E-04	1.99E-02	4/72 5.5%	61/14303 0.4%	*ALDH1A3 DHRS9 CYP1A1 CYP1B1*
34754	cellular hormone metabolic process	3.36E-04	2.30E-02	4/72 5.5%	66/14303 0.4%	*ALDH1A3 DHRS9 CYP1A1 CYP1B1*
9110	vitamin biosynthetic process	3.66E-04	2.30E-02	3/72 4.1%	28/14303 0.1%	*DHRS9 CYP1A1 CBLB*
6776	vitamin A metabolic process	4.07E-04	2.30E-02	3/72 4.1%	29/14303 0.2%	*ALDH1A3 DHRS9 CYP1A1*
1523	retinoid metabolic process	4.07E-04	2.30E-02	3/72 4.1%	29/14303 0.2%	*ALDH1A3 DHRS9 CYP1A1*
16101	diterpenoid metabolic process	4.07E-04	2.30E-02	3/72 4.1%	29/14303 0.2%	*ALDH1A3 DHRS9 CYP1A1*
7389	pattern specification process	4.18E-04	2.30E-02	7/72 9.7%	271/14303 1.8%	*FRAT1 BTG2 MFNG TSHZ1 FOXJ1 OTX1 GLI2*
2643	regulation of tolerance induction	5.16E-04	2.45E-02	2/72 2.7%	7/14303 0.0%	*FOXJ1 CBLB*
2645	positive regulation of tolerance induction	5.16E-04	2.45E-02	2/72 2.7%	7/14303 0.0%	*FOXJ1 CBLB*
9404	toxin metabolic process	5.16E-04	2.45E-02	2/72 2.7%	7/14303 0.0%	*CYP1A1 CYP1B1*
6766	vitamin metabolic process	7.33E-04	3.33E-02	4/72 5.5%	81/14303 0.5%	*ALDH1A3 DHRS9 CYP1A1 CBLB*
6721	terpenoid metabolic process	8.41E-04	3.46E-02	3/72 4.1%	37/14303 0.2%	*ALDH1A3 DHRS9 CYP1A1*
46685	response to arsenic	8.79E-04	3.46E-02	2/72 2.7%	9/14303 0.0%	*CDKN1A CYP1A1*
6775	fat-soluble vitamin metabolic process	9.82E-04	3.46E-02	3/72 4.1%	39/14303 0.2%	*ALDH1A3 DHRS9 CYP1A1*
32501	multicellular organismal process	9.95E-04	3.46E-02	35/72 48.6%	4375/14303 30.5%	*SPRR3 VIPR1 CDKN1A PTPRR BTG2 TSHZ1 KLK5 STC1 SECTM1 GLI2 FRAT1 GJA5 DPF1 SLITRK6 CYP1B1 OTX1 PRKCG VAV3 PLLP HTR1D LRRN4 ARID5B FOXJ1 NR0B2 APLN FOSL1 ALDH1A3 DHRS9 KRT16 MFNG*

						CYP1A1 PDE5A TAGLN3 LHX4 SPRR1B
6917	induction of apoptosis	1.02E-03	3.46E-02	7/72 9.7%	315/14303 2.2%	*VAV3 CDKN1A DPF1 TP53INP1 TNFSF10 BBC3 FGD2*
12502	induction of programmed cell death	1.04E-03	3.46E-02	7/72 9.7%	316/14303 2.2%	*VAV3 CDKN1A DPF1 TP53INP1 TNFSF10 BBC3 FGD2*
42981	regulation of apoptosis	1.04E-03	3.46E-02	12/72 16.6%	852/14303 5.9%	*FOSL1 VAV3 ALDH1A3 CDKN1A BTG2 DPF1 TP53INP1 TNFSF10 PDE5A LHX4 BBC3 FGD2*
48513	organ development	1.08E-03	3.46E-02	19/72 26.3%	1792/14303 12.5%	*VAV3 SPRR3 CDKN1A TSHZ1 KLK5 ARID5B SECTM1 NR0B2 GLI2 APLN ALDH1A3 DHRS9 KRT16 GJA5 CYP1A1 TAGLN3 OTX1 LHX4 SPRR1B*
7567	parturition	1.10E-03	3.46E-02	2/72 2.7%	10/14303 0.0%	*PLA2G4B CYP1A1*
55093	response to hyperoxia	1.10E-03	3.46E-02	2/72 2.7%	10/14303 0.0%	*CDKN1A CYP1A1*
43067	regulation of programmed cell death	1.12E-03	3.46E-02	12/72 16.6%	860/14303 6.0%	*FOSL1 VAV3 ALDH1A3 CDKN1A BTG2 DPF1 TP53INP1 TNFSF10 PDE5A LHX4 BBC3 FGD2*
10941	regulation of cell death	1.21E-03	3.60E-02	12/72 16.6%	867/14303 6.0%	*FOSL1 VAV3 ALDH1A3 CDKN1A BTG2 DPF1 TP53INP1 TNFSF10 PDE5A LHX4 BBC3 FGD2*
48731	system development	7.38E-06	7.71E-03	28/72 38.8%	2422/14303 16.9%	*SPRR3 CDKN1A PTPRR BTG2 TSHZ1 KLK5 STC1 SECTM1 GLI2 GJA5 DPF1 SLITRK6 OTX1 VAV3 PLLP LRRN4 ARID5B FOXJ1 NR0B2 APLN ALDH1A3 DHRS9 KRT16 CYP1A1 PDE5A TAGLN3 LHX4 SPRR1B*
48856	anatomical structure development	4.33E-05	1.18E-02	28/72 38.8%	2656/14303 18.5%	*SPRR3 CDKN1A PTPRR BTG2 TSHZ1 KLK5 STC1 SECTM1 GLI2 GJA5 DPF1 SLITRK6 OTX1 VAV3 PLLP LRRN4 ARID5B FOXJ1 NR0B2 APLN ALDH1A3 DHRS9 KRT16 CYP1A1 PDE5A TAGLN3*

						LHX4 SPRR1B
7275	multicellular organismal development	4.53E-05	1.18E-02	30/72 41.6%	2971/14303 20.7%	*SPRR3 CDKN1A PTPRR BTG2 TSHZ1 KLK5 STC1 SECTM1 GLI2 FRAT1 GJA5 DPF1 SLITRK6 OTX1 VAV3 PLLP LRRN4 ARID5B FOXJ1 NR0B2 APLN ALDH1A3 DHRS9 KRT16 MFNG CYP1A1 PDE5A TAGLN3 LHX4 SPRR1B*
43065	positive regulation of apoptosis	6.92E-05	1.18E-02	10/72 13.8%	442/14303 3.0%	*FOSL1 VAV3 ALDH1A3 CDKN1A DPF1 TP53INP1 TNFSF10 PDE5A BBC3 FGD2*
43068	positive regulation of programmed cell death	7.33E-05	1.18E-02	10/72 13.8%	445/14303 3.1%	*FOSL1 VAV3 ALDH1A3 CDKN1A DPF1 TP53INP1 TNFSF10 PDE5A BBC3 FGD2*
10942	positive regulation of cell death	7.89E-05	1.18E-02	10/72 13.8%	449/14303 3.1%	*FOSL1 VAV3 ALDH1A3 CDKN1A DPF1 TP53INP1 TNFSF10 PDE5A BBC3 FGD2*
42573	retinoic acid metabolic process	7.91E-05	1.18E-02	3/72 4.1%	17/14303 0.1%	*ALDH1A3 DHRS9 CYP1A1*
35238	vitamin A biosynthetic process	1.49E-04	1.56E-02	2/72 2.7%	4/14303 0.0%	*DHRS9 CYP1A1*
42904	9-cis-retinoic acid biosynthetic process	1.49E-04	1.56E-02	2/72 2.7%	4/14303 0.0%	*DHRS9 CYP1A1*
42905	9-cis-retinoic acid metabolic process	1.49E-04	1.56E-02	2/72 2.7%	4/14303 0.0%	*DHRS9 CYP1A1*
32502	developmental process	2.31E-04	1.99E-02	30/72 41.6%	3234/14303 22.6%	*SPRR3 CDKN1A PTPRR BTG2 TSHZ1 KLK5 STC1 SECTM1 GLI2 FRAT1 GJA5 DPF1 SLITRK6 OTX1 VAV3 PLLP LRRN4 ARID5B FOXJ1 NR0B2 APLN ALDH1A3 DHRS9 KRT16 MFNG CYP1A1 PDE5A TAGLN3 LHX4 SPRR1B*
42362	fat-soluble vitamin biosynthetic process	2.47E-04	1.99E-02	2/72 2.7%	5/14303 0.0%	*DHRS9 CYP1A1*
19748	secondary metabolic process	2.48E-04	1.99E-02	4/72 5.5%	61/14303 0.4%	*ALDH1A3 DHRS9 CYP1A1 CYP1B1*
34754	cellular hormone metabolic process	3.36E-04	2.30E-02	4/72 5.5%	66/14303 0.4%	*ALDH1A3 DHRS9 CYP1A1 CYP1B1*

9110	vitamin biosynthetic process	3.66E-04	2.30E-02	3/72 4.1%	28/14303 0.1%	*DHRS9 CYP1A1 CBLB*
6776	vitamin A metabolic process	4.07E-04	2.30E-02	3/72 4.1%	29/14303 0.2%	*ALDH1A3 DHRS9 CYP1A1*
1523	retinoid metabolic process	4.07E-04	2.30E-02	3/72 4.1%	29/14303 0.2%	*ALDH1A3 DHRS9 CYP1A1*
16101	diterpenoid metabolic process	4.07E-04	2.30E-02	3/72 4.1%	29/14303 0.2%	*ALDH1A3 DHRS9 CYP1A1*
7389	pattern specification process	4.18E-04	2.30E-02	7/72 9.7%	271/14303 1.8%	*FRAT1 BTG2 MFNG TSHZ1 FOXJ1 OTX1 GLI2*
2643	regulation of tolerance induction	5.16E-04	2.45E-02	2/72 2.7%	7/14303 0.0%	*FOXJ1 CBLB*
2645	positive regulation of tolerance induction	5.16E-04	2.45E-02	2/72 2.7%	7/14303 0.0%	*FOXJ1 CBLB*
9404	toxin metabolic process	5.16E-04	2.45E-02	2/72 2.7%	7/14303 0.0%	*CYP1A1 CYP1B1*
6766	vitamin metabolic process	7.33E-04	3.33E-02	4/72 5.5%	81/14303 0.5%	*ALDH1A3 DHRS9 CYP1A1 CBLB*
6721	terpenoid metabolic process	8.41E-04	3.46E-02	3/72 4.1%	37/14303 0.2%	*ALDH1A3 DHRS9 CYP1A1*
46685	response to arsenic	8.79E-04	3.46E-02	2/72 2.7%	9/14303 0.0%	*CDKN1A CYP1A1*
6775	fat-soluble vitamin metabolic process	9.82E-04	3.46E-02	3/72 4.1%	39/14303 0.2%	*ALDH1A3 DHRS9 CYP1A1*
32501	multicellular organismal process	9.95E-04	3.46E-02	35/72 48.6%	4375/14303 30.5%	*SPRR3 VIPR1 CDKN1A PTPRR BTG2 TSHZ1 KLK5 STC1 SECTM1 GLI2 FRAT1 GJA5 DPF1 SLITRK6 CYP1B1 OTX1 PRKCG VAV3 PLLP HTR1D LRRN4 ARID5B FOXJ1 NR0B2 APLN FOSL1 ALDH1A3 DHRS9 KRT16 MFNG CYP1A1 PDE5A TAGLN3 LHX4 SPRR1B*
6917	induction of apoptosis	1.02E-03	3.46E-02	7/72 9.7%	315/14303 2.2%	*VAV3 CDKN1A DPF1 TP53INP1 TNFSF10 BBC3 FGD2*
12502	induction of programmed cell death	1.04E-03	3.46E-02	7/72 9.7%	316/14303 2.2%	*VAV3 CDKN1A DPF1 TP53INP1 TNFSF10 BBC3 FGD2*
42981	regulation of apoptosis	1.04E-03	3.46E-02	12/72 16.6%	852/14303 5.9%	*FOSL1 VAV3 ALDH1A3 CDKN1A BTG2*

						DPF1 TP53INP1 TNFSF10 PDE5A LHX4 BBC3 FGD2
48513	organ development	1.08E-03	3.46E-02	19/72 26.3%	1792/14303 12.5%	*VAV3 SPRR3 CDKN1A TSHZ1 KLK5 ARID5B SECTM1 NR0B2 GLI2 APLN ALDH1A3 DHRS9 KRT16 GJA5 CYP1A1 TAGLN3 OTX1 LHX4 SPRR1B*
7567	parturition	1.10E-03	3.46E-02	2/72 2.7%	10/14303 0.0%	*PLA2G4B CYP1A1*
55093	response to hyperoxia	1.10E-03	3.46E-02	2/72 2.7%	10/14303 0.0%	*CDKN1A CYP1A1*
43067	regulation of programmed cell death	1.12E-03	3.46E-02	12/72 16.6%	860/14303 6.0%	*FOSL1 VAV3 ALDH1A3 CDKN1A BTG2 DPF1 TP53INP1 TNFSF10 PDE5A LHX4 BBC3 FGD2*
10941	regulation of cell death	1.21E-03	3.60E-02	12/72 16.6%	867/14303 6.0%	*FOSL1 VAV3 ALDH1A3 CDKN1A BTG2 DPF1 TP53INP1 TNFSF10 PDE5A LHX4 BBC3 FGD2*
48731	system development	7.38E-06	7.71E-03	28/72 38.8%	2422/14303 16.9%	*SPRR3 CDKN1A PTPRR BTG2 TSHZ1 KLK5 STC1 SECTM1 GLI2 GJA5 DPF1 SLITRK6 OTX1 VAV3 PLLP LRRN4 ARID5B FOXJ1 NR0B2 APLN ALDH1A3 DHRS9 KRT16 CYP1A1 PDE5A TAGLN3 LHX4 SPRR1B*
48856	anatomical structure development	4.33E-05	1.18E-02	28/72 38.8%	2656/14303 18.5%	*SPRR3 CDKN1A PTPRR BTG2 TSHZ1 KLK5 STC1 SECTM1 GLI2 GJA5 DPF1 SLITRK6 OTX1 VAV3 PLLP LRRN4 ARID5B FOXJ1 NR0B2 APLN ALDH1A3 DHRS9 KRT16 CYP1A1 PDE5A TAGLN3 LHX4 SPRR1B*
7275	multicellular organismal development	4.53E-05	1.18E-02	30/72 41.6%	2971/14303 20.7%	*SPRR3 CDKN1A PTPRR BTG2 TSHZ1 KLK5 STC1 SECTM1 GLI2 FRAT1 GJA5 DPF1 SLITRK6 OTX1 VAV3 PLLP LRRN4 ARID5B FOXJ1 NR0B2 APLN ALDH1A3 DHRS9 KRT16 MFNG CYP1A1 PDE5A TAGLN3 LHX4 SPRR1B*

43065	positive regulation of apoptosis	6.92E-05	1.18E-02	10/72 13.8%	442/14303 3.0%	*FOSL1 VAV3 ALDH1A3 CDKN1A DPF1 TP53INP1 TNFSF10 PDE5A BBC3 FGD2*
43068	positive regulation of programmed cell death	7.33E-05	1.18E-02	10/72 13.8%	445/14303 3.1%	*FOSL1 VAV3 ALDH1A3 CDKN1A DPF1 TP53INP1 TNFSF10 PDE5A BBC3 FGD2*
10942	positive regulation of cell death	7.89E-05	1.18E-02	10/72 13.8%	449/14303 3.1%	*FOSL1 VAV3 ALDH1A3 CDKN1A DPF1 TP53INP1 TNFSF10 PDE5A BBC3 FGD2*
42573	retinoic acid metabolic process	7.91E-05	1.18E-02	3/72 4.1%	17/14303 0.1%	*ALDH1A3 DHRS9 CYP1A1*
35238	vitamin A biosynthetic process	1.49E-04	1.56E-02	2/72 2.7%	4/14303 0.0%	*DHRS9 CYP1A1*
42904	9-cis-retinoic acid biosynthetic process	1.49E-04	1.56E-02	2/72 2.7%	4/14303 0.0%	*DHRS9 CYP1A1*
42905	9-cis-retinoic acid metabolic process	1.49E-04	1.56E-02	2/72 2.7%	4/14303 0.0%	*DHRS9 CYP1A1*
32502	developmental process	2.31E-04	1.99E-02	30/72 41.6%	3234/14303 22.6%	*SPRR3 CDKN1A PTPRR BTG2 TSHZ1 KLK5 STC1 SECTM1 GLI2 FRAT1 GJA5 DPF1 SLITRK6 OTX1 VAV3 PLLP LRRN4 ARID5B FOXJ1 NR0B2 APLN ALDH1A3 DHRS9 KRT16 MFNG CYP1A1 PDE5A TAGLN3 LHX4 SPRR1B*
42362	fat-soluble vitamin biosynthetic process	2.47E-04	1.99E-02	2/72 2.7%	5/14303 0.0%	*DHRS9 CYP1A1*
19748	secondary metabolic process	2.48E-04	1.99E-02	4/72 5.5%	61/14303 0.4%	*ALDH1A3 DHRS9 CYP1A1 CYP1B1*
34754	cellular hormone metabolic process	3.36E-04	2.30E-02	4/72 5.5%	66/14303 0.4%	*ALDH1A3 DHRS9 CYP1A1 CYP1B1*
9110	vitamin biosynthetic process	3.66E-04	2.30E-02	3/72 4.1%	28/14303 0.1%	*DHRS9 CYP1A1 CBLB*
6776	vitamin A metabolic process	4.07E-04	2.30E-02	3/72 4.1%	29/14303 0.2%	*ALDH1A3 DHRS9 CYP1A1*
1523	retinoid metabolic process	4.07E-04	2.30E-02	3/72 4.1%	29/14303 0.2%	*ALDH1A3 DHRS9 CYP1A1*
16101	diterpenoid metabolic process	4.07E-04	2.30E-02	3/72 4.1%	29/14303 0.2%	*ALDH1A3 DHRS9 CYP1A1*
7389	pattern specification process	4.18E-04	2.30E-02	7/72 9.7%	271/14303 1.8%	*FRAT1 BTG2 MFNG TSHZ1 FOXJ1 OTX1 GLI2*

2643	regulation of tolerance induction	5.16E-04	2.45E-02	2/72 2.7%	7/14303 0.0%	*FOXJ1 CBLB*
2645	positive regulation of tolerance induction	5.16E-04	2.45E-02	2/72 2.7%	7/14303 0.0%	*FOXJ1 CBLB*
9404	toxin metabolic process	5.16E-04	2.45E-02	2/72 2.7%	7/14303 0.0%	*CYP1A1 CYP1B1*
6766	vitamin metabolic process	7.33E-04	3.33E-02	4/72 5.5%	81/14303 0.5%	*ALDH1A3 DHRS9 CYP1A1 CBLB*
6721	terpenoid metabolic process	8.41E-04	3.46E-02	3/72 4.1%	37/14303 0.2%	*ALDH1A3 DHRS9 CYP1A1*
46685	response to arsenic	8.79E-04	3.46E-02	2/72 2.7%	9/14303 0.0%	*CDKN1A CYP1A1*
6775	fat-soluble vitamin metabolic process	9.82E-04	3.46E-02	3/72 4.1%	39/14303 0.2%	*ALDH1A3 DHRS9 CYP1A1*
32501	multicellular organismal process	9.95E-04	3.46E-02	35/72 48.6%	4375/14303 30.5%	*SPRR3 VIPR1 CDKN1A PTPRR BTG2 TSHZ1 KLK5 STC1 SECTM1 GLI2 FRAT1 GJA5 DPF1 SLITRK6 CYP1B1 OTX1 PRKCG VAV3 PLLP HTR1D LRRN4 ARID5B FOXJ1 NR0B2 APLN FOSL1 ALDH1A3 DHRS9 KRT16 MFNG CYP1A1 PDE5A TAGLN3 LHX4 SPRR1B*
6917	induction of apoptosis	1.02E-03	3.46E-02	7/72 9.7%	315/14303 2.2%	*VAV3 CDKN1A DPF1 TP53INP1 TNFSF10 BBC3 FGD2*
12502	induction of programmed cell death	1.04E-03	3.46E-02	7/72 9.7%	316/14303 2.2%	*VAV3 CDKN1A DPF1 TP53INP1 TNFSF10 BBC3 FGD2*
42981	regulation of apoptosis	1.04E-03	3.46E-02	12/72 16.6%	852/14303 5.9%	*FOSL1 VAV3 ALDH1A3 CDKN1A BTG2 DPF1 TP53INP1 TNFSF10 PDE5A LHX4 BBC3 FGD2*
48513	organ development	1.08E-03	3.46E-02	19/72 26.3%	1792/14303 12.5%	*VAV3 SPRR3 CDKN1A TSHZ1 KLK5 ARID5B SECTM1 NR0B2 GLI2 APLN ALDH1A3 DHRS9 KRT16 GJA5 CYP1A1 TAGLN3 OTX1 LHX4 SPRR1B*
7567	parturition	1.10E-03	3.46E-02	2/72 2.7%	10/14303 0.0%	*PLA2G4B CYP1A1*

55093	response to hyperoxia	1.10E-03	3.46E-02	2/72 2.7%	10/14303 0.0%	*CDKN1A CYP1A1*
43067	regulation of programmed cell death	1.12E-03	3.46E-02	12/72 16.6%	860/14303 6.0%	*FOSL1 VAV3 ALDH1A3 CDKN1A BTG2 DPF1 TP53INP1 TNFSF10 PDE5A LHX4 BBC3 FGD2*
10941	regulation of cell death	1.21E-03	3.60E-02	12/72 16.6%	867/14303 6.0%	*FOSL1 VAV3 ALDH1A3 CDKN1A BTG2 DPF1 TP53INP1 TNFSF10 PDE5A LHX4 BBC3 FGD2*
43065	positive regulation of apoptosis	6.92E-05	1.18E-02	10/72 13.8%	442/14303 3.0%	*FOSL1 VAV3 ALDH1A3 CDKN1A DPF1 TP53INP1 TNFSF10 PDE5A BBC3 FGD2*
43068	positive regulation of programmed cell death	7.33E-05	1.18E-02	10/72 13.8%	445/14303 3.1%	*FOSL1 VAV3 ALDH1A3 CDKN1A DPF1 TP53INP1 TNFSF10 PDE5A BBC3 FGD2*
10942	positive regulation of cell death	7.89E-05	1.18E-02	10/72 13.8%	449/14303 3.1%	*FOSL1 VAV3 ALDH1A3 CDKN1A DPF1 TP53INP1 TNFSF10 PDE5A BBC3 FGD2*
42573	retinoic acid metabolic process	7.91E-05	1.18E-02	3/72 4.1%	17/14303 0.1%	*ALDH1A3 DHRS9 CYP1A1*
35238	vitamin A biosynthetic process	1.49E-04	1.56E-02	2/72 2.7%	4/14303 0.0%	*DHRS9 CYP1A1*
42904	9-cis-retinoic acid biosynthetic process	1.49E-04	1.56E-02	2/72 2.7%	4/14303 0.0%	*DHRS9 CYP1A1*
42905	9-cis-retinoic acid metabolic process	1.49E-04	1.56E-02	2/72 2.7%	4/14303 0.0%	*DHRS9 CYP1A1*

[a] Corrected *p*-value: After correction, the *p* value in the hypergeometric test

[b] Cluster frequency: the numerator represents the number of each GO term genes and the denominator represents the total number of genes with GO annotation.

[c] Total frequency: the numerator represents the number of reference genes annotated in the listed GO term and the denominator represents the number of reference genes with GO annotation.

Table S7 Gene ontology (GO) enrichment analysis of 147 differentially expressed genes (DEGs) between wild-type-spore treated HT-29 cells and control

GO ID	GO Description	*p*-value	Corrected *p*-value[a]	Cluster frequency[b]	Total frequency[c]	Genes
6941	striated muscle contraction	4.84E-05	1.33E-02	3/27 11.1%	38/14305 0.2%	*RYR2 TNNC2 TCAP*
45595	regulation of cell differentiation	5.62E-05	1.33E-02	7/27 25.9%	553/14305 3.8%	*SOCS3 CCDC88A CITED1 PLA2G2A SPP1 NPHP3 HOPX*
45596	negative regulation of cell differentiation	6.75E-05	1.33E-02	5/27 18.5%	234/14305 1.6%	*CITED1 PLA2G2A SPP1 NPHP3 HOPX*
50793	regulation of developmental process	7.31E-05	1.33E-02	8/27 29.6%	791/14305 5.5%	*SOCS3 CCDC88A FOXD1 CITED1 PLA2G2A SPP1 NPHP3 HOPX*
90183	regulation of kidney development	9.54E-05	1.39E-02	2/27 7.4%	8/14305 0.0%	*FOXD1 CITED1*
60712	spongiotrophoblast layer development	1.23E-04	1.49E-02	2/27 7.4%	9/14305 0.0%	*SOCS3 CITED1*
51093	negative regulation of developmental process	1.82E-04	1.70E-02	5/27 18.5%	289/14305 2.0%	*CITED1 PLA2G2A SPP1 NPHP3 HOPX*
6936	muscle contraction	1.87E-04	1.70E-02	4/27 14.8%	154/14305 1.0%	*RYR2 CALD1 TNNC2 TCAP*
3012	muscle system process	2.61E-04	1.92E-02	4/27 14.8%	168/14305 1.1%	*RYR2 CALD1 TNNC2 TCAP*
3009	skeletal muscle contraction	2.64E-04	1.92E-02	2/27 7.4%	13/14305 0.0%	*TNNC2 TCAP*
60048	cardiac muscle contraction	4.59E-04	2.79E-02	2/27 7.4%	17/14305 0.1%	*RYR2 TCAP*
9605	response to external stimulus	4.71E-04	2.79E-02	6/27 22.2%	551/14305 3.8%	*RYR2 SOCS3 CXCR3 NEURL1 SPP1 TCAP*
1890	placenta development	5.72E-04	2.79E-02	3/27 11.1%	87/14305 0.6%	*SOCS3 CITED1 SPP1*
30154	cell differentiation	5.85E-04	2.79E-02	10/27 37.0%	1668/14305 11.6%	*SPTBN4 SOCS3 CCDC88A CITED1 PLA2G2A NEURL1 SPP1 TCAP SH2B2 HOPX*
60047	heart contraction	7.05E-04	2.79E-02	2/27 7.4%	21/14305 0.1%	*RYR2 TCAP*

50879	multicellular organismal movement	7.05E-04	2.79E-02	2/27 7.4%	21/14305 0.1%	*TNNC2 TCAP*
50881	musculoskeletal movement	7.05E-04	2.79E-02	2/27 7.4%	21/14305 0.1%	*TNNC2 TCAP*
3015	heart process	7.05E-04	2.79E-02	2/27 7.4%	21/14305 0.1%	*RYR2 TCAP*
48869	cellular developmental process	7.28E-04	2.79E-02	10/27 37.0%	1714/14305 11.9%	*SPTBN4 SOCS3 CCDC88A CITED1 PLA2G2A NEURL1 SPP1 TCAP SH2B2 HOPX*
61138	morphogenesis of a branching epithelium	8.58E-04	2.80E-02	3/27 11.1%	100/14305 0.6%	*SOCS3 FOXD1 CITED1*
1658	branching involved in ureteric bud morphogenesis	1.26E-03	2.80E-02	2/27 7.4%	28/14305 0.1%	*FOXD1 CITED1*
7010	cytoskeleton organization	1.34E-03	2.80E-02	5/27 18.5%	448/14305 3.1%	*SPTBN4 CALD1 NEURL1 TCAP SH2B2*
60675	ureteric bud morphogenesis	1.44E-03	2.80E-02	2/27 7.4%	30/14305 0.2%	*FOXD1 CITED1*
1763	morphogenesis of a branching structure	1.49E-03	2.80E-02	3/27 11.1%	121/14305 0.8%	*SOCS3 FOXD1 CITED1*
10927	cellular component assembly involved in morphogenesis	1.54E-03	2.80E-02	2/27 7.4%	31/14305 0.2%	*NEURL1 TCAP*
32868	response to insulin stimulus	1.56E-03	2.80E-02	3/27 11.1%	123/14305 0.8%	*SOCS3 CITED1 SH2B2*
60711	labyrinthine layer development	1.64E-03	2.80E-02	2/27 7.4%	32/14305 0.2%	*SOCS3 CITED1*
48729	tissue morphogenesis	1.65E-03	2.80E-02	4/27 14.8%	275/14305 1.9%	*SOCS3 FOXD1 CITED1 TCAP*
6805	xenobiotic metabolic process	1.75E-03	2.80E-02	2/27 7.4%	33/14305 0.2%	*UGT2B15 SULT1A3*
71466	cellular response to xenobiotic stimulus	1.75E-03	2.80E-02	2/27 7.4%	33/14305 0.2%	*UGT2B15 SULT1A3*
48856	anatomical structure development	1.77E-03	2.80E-02	12/27 44.4%	2656/14305 18.5%	*SPTBN4 SOCS3 CCDC88A FOXD1 CITED1 PLA2G2A NEURL1 SPP1 NPHP3 TCAP SH2B2 HOPX*

60678	dichotomous subdivision of terminal units involved in ureteric bud branching	1.89E-03	2.80E-02	1/27 3.7%	1/14305 0.0%	*FOXD1*
3339	regulation of mesenchymal to epithelial transition involved in metanephros morphogenesis	1.89E-03	2.80E-02	1/27 3.7%	1/14305 0.0%	*CITED1*
3340	negative regulation of mesenchymal to epithelial transition involved in metanephros morphogenesis	1.89E-03	2.80E-02	1/27 3.7%	1/14305 0.0%	*CITED1*
8627	induction of apoptosis by ionic changes	1.89E-03	2.80E-02	1/27 3.7%	1/14305 0.0%	*RYR2*
72127	renal capsule development	1.89E-03	2.80E-02	1/27 3.7%	1/14305 0.0%	*FOXD1*
71105	response to interleukin-11	1.89E-03	2.80E-02	1/27 3.7%	1/14305 0.0%	*CITED1*
72129	renal capsule formation	1.89E-03	2.80E-02	1/27 3.7%	1/14305 0.0%	*FOXD1*
71104	response to interleukin-9	1.89E-03	2.80E-02	1/27 3.7%	1/14305 0.0%	*CITED1*
72128	renal capsule morphogenesis	1.89E-03	2.80E-02	1/27 3.7%	1/14305 0.0%	*FOXD1*
71107	response to parathyroid hormone stimulus	1.89E-03	2.80E-02	1/27 3.7%	1/14305 0.0%	*CITED1*
72130	renal capsule specification	1.89E-03	2.80E-02	1/27 3.7%	1/14305 0.0%	*FOXD1*
72213	metanephric capsule development	1.89E-03	2.80E-02	1/27 3.7%	1/14305 0.0%	*FOXD1*
48683	regulation of collateral sprouting of intact axon in response to injury	1.89E-03	2.80E-02	1/27 3.7%	1/14305 0.0%	*SPP1*
48685	negative regulation of collateral sprouting of intact axon in response to injury	1.89E-03	2.80E-02	1/27 3.7%	1/14305 0.0%	*SPP1*

72265	metanephric capsule morphogenesis	1.89E-03	2.80E-02	1/27 3.7%	1/14305 0.0%	*FOXD1*
72267	metanephric capsule specification	1.89E-03	2.80E-02	1/27 3.7%	1/14305 0.0%	*FOXD1*
72266	metanephric capsule formation	1.89E-03	2.80E-02	1/27 3.7%	1/14305 0.0%	*FOXD1*
1922	B-1 B cell homeostasis	1.89E-03	2.80E-02	1/27 3.7%	1/14305 0.0%	*SH2B2*
1656	metanephros development	1.96E-03	2.86E-02	2/27 7.4%	35/14305 0.2%	*FOXD1 CITED1*
9410	response to xenobiotic stimulus	2.19E-03	3.07E-02	2/27 7.4%	37/14305 0.2%	*UGT2B15 SULT1A3*
90100	positive regulation of transmembrane receptor protein serine/threonine kinase signaling pathway	2.19E-03	3.07E-02	2/27 7.4%	37/14305 0.2%	*FOXD1 CITED1*
50896	response to stimulus	2.85E-03	3.91E-02	14/27 51.8%	3633/14305 25.3%	*RYR2 SPTBN4 CITED1 PLA2G4B UGT2B15 PLA2G2A SOCS3 CXCR3 NEURL1 SPP1 TCAP HLA-DOB SULT1A3 SH2B2*
51239	regulation of multicellular organismal process	2.97E-03	4.01E-02	7/27 25.9%	1067/14305 7.4%	*RYR2 SPTBN4 CCDC88A FOXD1 CITED1 TNNC2 SPP1*
70669	response to interleukin-2	3.77E-03	4.58E-02	1/27 3.7%	2/14305 0.0%	*CITED1*
14808	release of sequestered calcium ion into cytosol by sarcoplasmic reticulum	3.77E-03	4.58E-02	1/27 3.7%	2/14305 0.0%	*RYR2*
72215	regulation of metanephros development	3.77E-03	4.58E-02	1/27 3.7%	2/14305 0.0%	*CITED1*
48670	regulation of collateral sprouting	3.77E-03	4.58E-02	1/27 3.7%	2/14305 0.0%	*SPP1*
48671	negative regulation of collateral sprouting	3.77E-03	4.58E-02	1/27 3.7%	2/14305 0.0%	*SPP1*
10881	regulation of cardiac muscle contraction by regulation of the	3.77E-03	4.58E-02	1/27 3.7%	2/14305 0.0%	*RYR2*

	release of sequestered calcium ion					
32989	cellular component morphogenesis	4.04E-03	4.82E-02	4/27 14.8%	352/14305 2.4%	*SPTBN4 CITED1 NEURL1 TCAP*

[a] Corrected *p*-value: After correction, the *p* value in the hypergeometric test

[b] Cluster frequency: the numerator represents the number of each GO term genes and the denominator represents the total number of genes with GO annotation.

[c] Total frequency: the numerator represents the number of reference genes annotated in the listed GO term and the denominator represents the number of reference genes with GO annotation

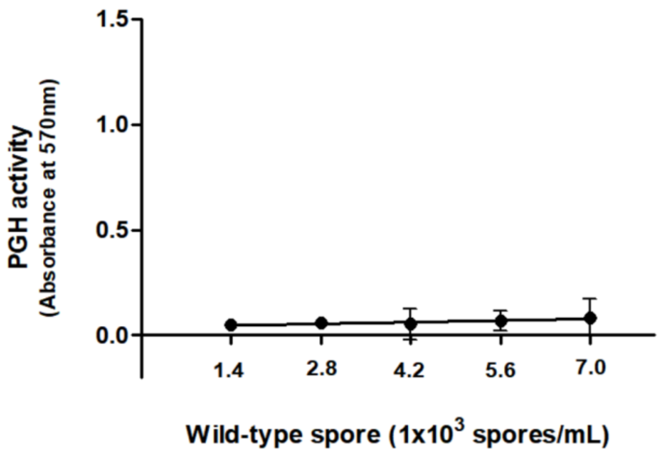

Fig. S1 Determination of the peptidoglycan hydrolase activity of wild-type spore. After treatment of peptidoglycan with different concentrations of wild-type spore at 37℃ for 15 min, the absorbance of each sample was measured at 570 nm. All tests were performed in triplicate, and the data are presented as mean ± standard deviation.

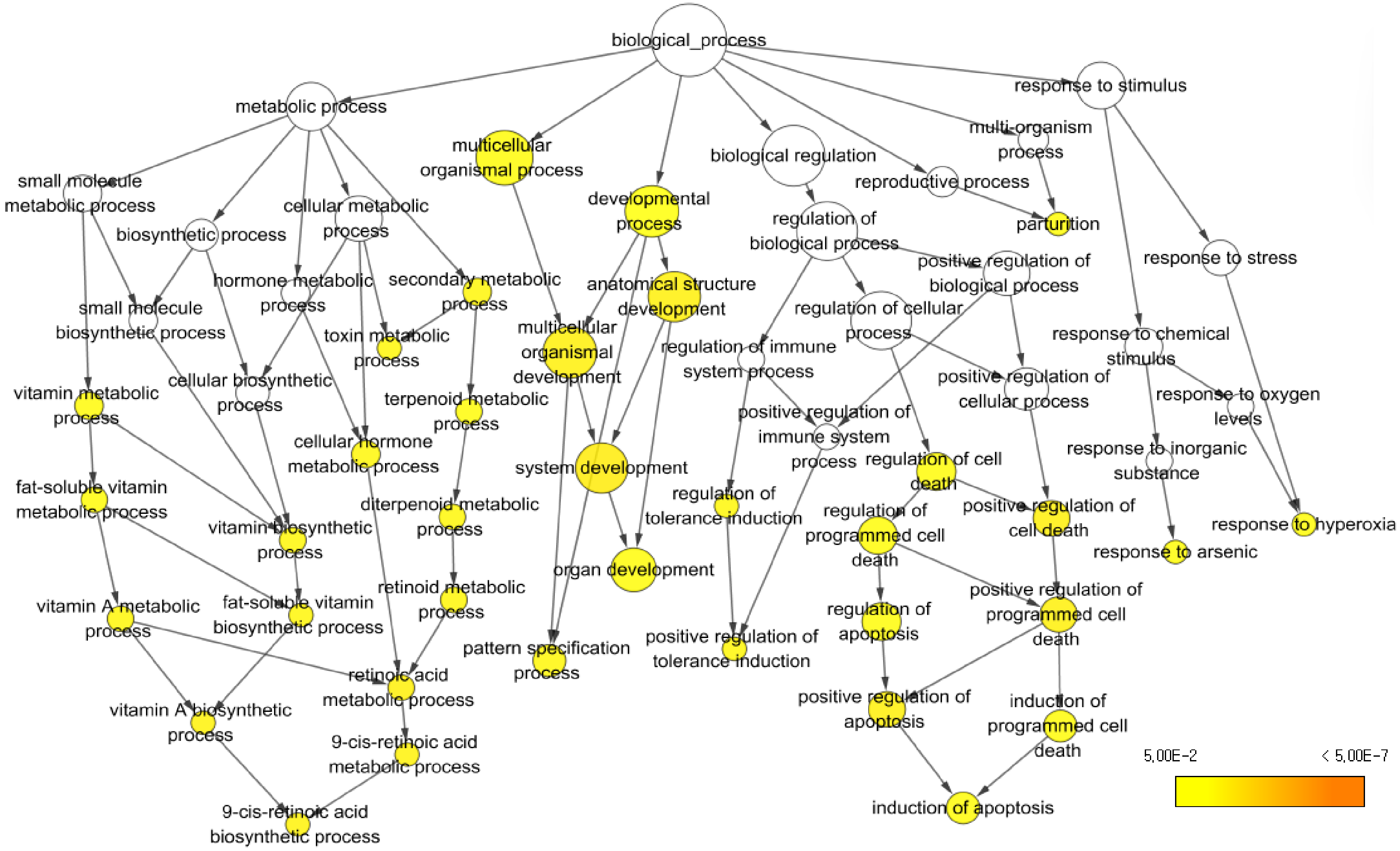

Fig. S2 Gene ontology (GO) analysis for DEGs between CotG-p40- and wild-type spore-treated HT-29 cells

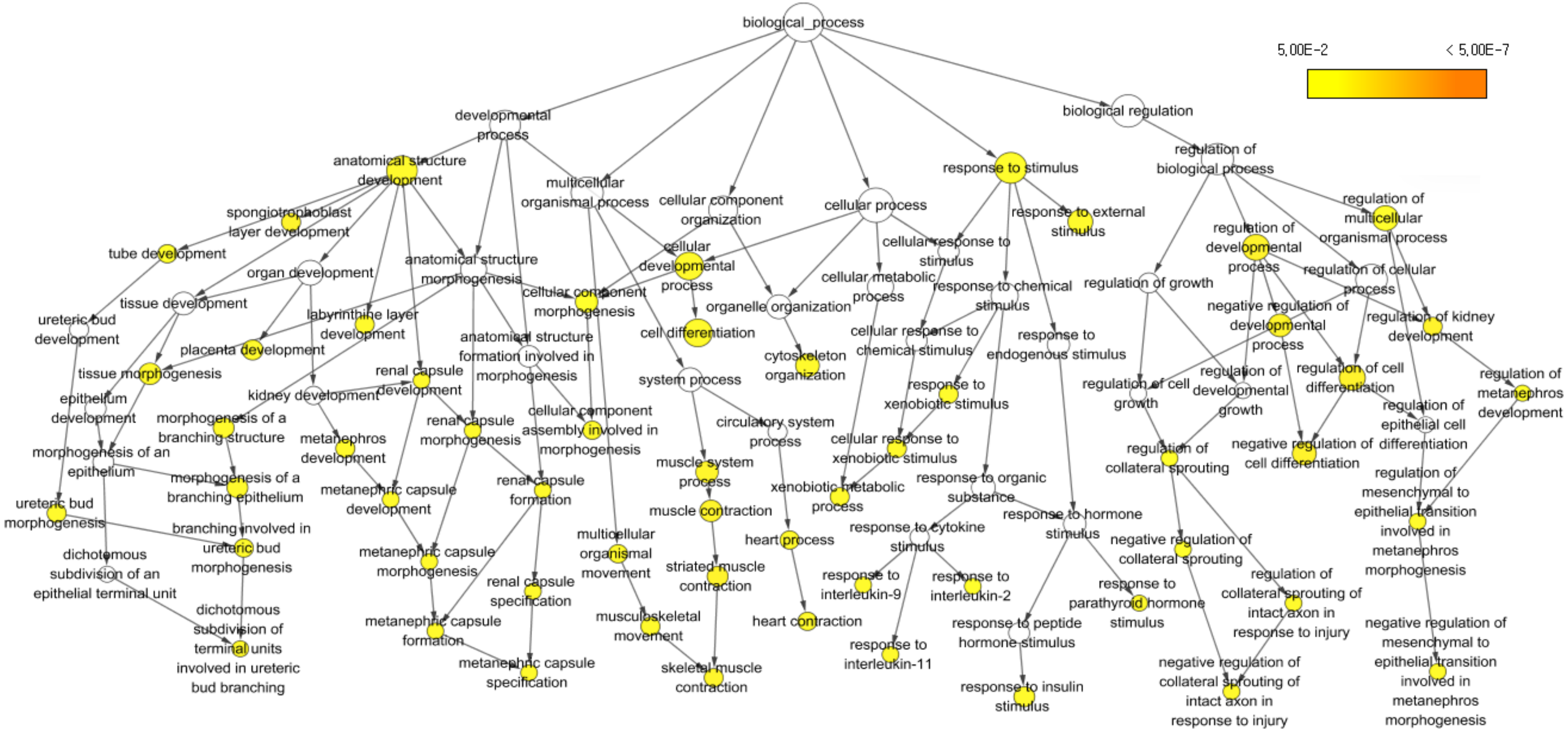

Fig. S3 Gene ontology (GO) analysis for DEGs between wild-type spore-treated HT-29 cells and control
